# Mobile nudges and financial incentives to improve coverage of timely neonatal vaccination in rural areas (GEVaP trial): A 3-armed cluster randomized controlled trial in Northern Ghana

**DOI:** 10.1371/journal.pone.0247485

**Published:** 2021-05-19

**Authors:** Gillian Levine, Amadu Salifu, Issah Mohammed, Günther Fink

**Affiliations:** 1 Department of Epidemiology and Public Health, Swiss Tropical and Public Health Institute, Basel, Switzerland; 2 University of Basel, Basel, Switzerland; 3 Innovations for Poverty Action, Tamale, Ghana; IAVI, UNITED STATES

## Abstract

**Background:**

Despite progress in vaccination coverage, timeliness of childhood vaccination remains a challenge in many settings. We aimed to assess if mobile phone-based reminders and incentives to health workers and caregivers could increase timely neonatal vaccination in a rural, low-resource setting.

**Methods:**

We conducted an open-label cluster randomized controlled 1:1:1 trial with three arms in 15 communities in Northern Ghana. Communities were randomized to 1) a voice call reminder intervention; 2) a community health volunteer (CHV) intervention with incentivized rewards; 3) control. In the voice call reminder arm, a study staff member made voice calls to mothers shortly after birth to encourage vaccination and provide personalized information about available vaccination services. In the incentive arm, CHVs promoted infant vaccination and informed women with recent births about available vaccination opportunities. Both CHVs and women were provided small monetary incentives for on-time early infant vaccination in this arm, delivered using mobile phone-based banking applications. No study activities were conducted in control communities. A population-based survey compared vaccination coverage across arms in the pre-intervention and intervention periods. The primary endpoint was completion of at least one dose of Polio vaccine within 14 days of life and BCG vaccination within 28 days of life.

**Results:**

Six-hundred ninety births were identified; 106, 88, and 88 from pre-intervention and 150, 135, and 123 in the intervention period, in the control, voice call reminder and CHV incentive arms, respectively. In adjusted intent-to-treat analysis, voice call reminders were associated with 10.5 percentage point (95% CI: 4.0, 17.1) higher coverage of on-time vaccination, while mobile phone-based incentives were associated with 49.5 percentage point (95% CI: 26.4, 72.5) higher coverage.

**Conclusion:**

Community-based interventions using mobile phone technologies can improve timely early vaccination coverage. A CHV approach with incentives to community workers and caregivers was a more effective strategy than voice call reminders. The impact of vaccination “nudges” via voice calls may be constrained in settings where network coverage and phone ownership are limited.

**Trial registration:**

This trial was registered at ClinicalTrials.gov; NCT03797950.

## Introduction

Despite progress in overall vaccination coverage, timeliness of childhood vaccines remains a challenge in many settings [1,2]. Delays and gaps in early vaccination leave young infants at risk of preventable infections and severe morbidity and mortality. The World Health Organization (WHO) recommends that the first dose of Polio and Bacillus Calmette–Guérin (BCG) vaccinations should be administered as soon as possible after birth, and within the first 14 and 28 days of life, respectively [3]. In many settings these vaccinations are part of the routine services delivered in health facilities at the time of childbirth.

Although access to health facilities has improved and facility-based delivery coverage has increased in many low-resource settings, disparities and barriers to access and utilization remain [4]. At the same time, access to mobile phones and mobile data network coverage is expanding rapidly. Sub-Saharan Africa is the fastest-growing mobile market in the world, and approximately 80% of adults and the majority of households now have a mobile phone [5,6]. Mobile phone-based public health (mHealth) strategies offer opportunities to reach difficult to reach populations with information and messaging, to improve awareness, demand for and utilization of public health services, and offer simple and low-cost strategies for supporting communication, coordination, tracking and supervision of public health programs. Text message reminders can improve service utilization, attendance at scheduled health visits, and medication adherence for acute and chronic illness [7–9], and have largely been found to be cost-effective [10]. Text messages have also demonstrated effectiveness for support and supervision of health workers and improving coordination of community-based delivery strategies. Evidence on the influence of mobile phone-based reminders on preventive health behaviors is more limited; few studies have evaluated the impact on preventive health services, or utilization of child health services in low-income settings [11,12]. Text messages can be automated and standardized, and are thus low-cost and relatively simple to implement. However, in populations with limited literacy and numeracy, or when phone sharing among household or community members is common, text messages may be a less effective strategy. Additionally, if a lack of awareness as to when, where and how to access services is a key barrier to service utilization, automated or standardized text messages may be ineffective. In such settings, personalized voice calls to provide tailored information and inform clients of service access points may be a more appropriate and effective intervention approach, albeit more expensive and complex.

Financial incentives have also demonstrated impact in improving health worker performance and in influencing individual behavior-change in public health programs. There is a paucity of research on the impact of incentives that target both health workers and clients on the utilization of child health preventive services in sub-Saharan Africa.

To assess the extent to which mobile phone-based reminders and incentives to community health workers and caregivers could increase population-level timely neonatal vaccination in rural, low-resource settings, we conducted a small cluster randomized trial.

## Methods

We conducted an open-label cluster randomized controlled trial with three arms in 15 communities (clusters) in Northern Ghana. Communities were randomized to three groups: 1) a voice call reminder intervention (Intervention Group A, 5 communities); 2) a CHV intervention with incentivized rewards (Intervention Group B, 5 communities); 3) control (5 communities). A cluster randomized design was used for evaluation because the intervention was fundamentally delivered at the cluster level by local community volunteers, and thus individual randomization was not feasible. A checklist of criteria for compliance with the Consort Statement for cluster randomized controlled trials is included (S1 File).

### Setting and population

The Ghana Early Vaccination Program (GEVaP) study was conducted in rural Karaga District in Ghana’s Northern Region. The population in the region generally has substantially worse socioeconomic and health status than the country overall. Approximately 66% of women 15–49 in the region have never attended school, as opposed to 19% on average nationally; 49% have not accessed any media sources (tv, newspaper or magazine or radio) in the previous week, compared with 31% on average nationally. Approximately 35% of women in the Northern Region delivered their last child in a health facility, whereas nationally 73% on average are delivered in facilities. Preventive child health services including vaccinations are coordinated by the Ghana Health Services (GHS) and District Authorities and are available at selected facilities via fixed clinics or via “outreach” services delivered to communities at routine intervals. Vaccinations are usually offered at fixed clinics only during designated days and times at facilities, usually on a weekly or twice-weekly basis. “Outreach” vaccination services are typically delivered once per month in communities. Child births commonly occur outside of health facilities [13]. Although 97% of children in the region receive BCG vaccination by two years of life, less than half are vaccinated within the recommended first month of life. Less than 60% of children in the region receive the first dose of Polio vaccination and only 41% receive all age-appropriate vaccinations by two years of life [13]. One major barrier to timely vaccination in the region is timely and complete birth registration. Community Health Volunteers (CHV) trained by the GHS are tasked with documenting births, providing health promotion and health education activities in their communities, but are not a formal cadre within the GHS and are not remunerated. As a result, few births are reported to or officially registered with local authorities; registration is often delayed and civil registration systems are rarely or incompletely linked with health service information systems [13,14].

### Interventions

In each intervention community, teams of 2–4 CHVs previously selected and trained by the GHS for health and development work were invited to participate in the GEVaP program. If no previously trained CHVs were available, community leaders were asked to appoint new CHVs for the program. CHVs received standardized training. Many CHVs were not able to read and to write, thus training, tools and documentation materials were adapted to be appropriate for limited literacy and numeracy. CHVs were expected to document and report births in their communities within a week to a central study personnel and were provided with mobile network/data credit via “mobile money” to cover the costs of communicating with and sending verification materials to study staff. CHVs were given a 2 cedis reward (USD 0.50) for each birth reported within the first week of life. Vaccine records booklets were distributed to GHS staff in intervention and control communities to support documentation of services. A TiDIER checklist listing the location of items describing intervention components is included (S2 File) [15].

#### Intervention Arm A

In intervention arm A (“voice call reminders”) CHVs documented and reported births to study personnel as described above. CHVs invited caregivers who had delivered a child in the previous week to participate in the program and obtained informed consent from eligible women. Enrolled women provided a phone number at which they could be contacted, either a personal or shared phone or phone to which they had access. CHVs were not instructed to provide specific follow-up to caregivers in this arm to encourage or track vaccination after enrollment, in addition to any contact or services delivered as part of their routine community health activities. A central study staff made voice calls to congratulate caregivers on the birth, highlighted the importance of early vaccinations and provide information on where and when vaccine services were available for young infants in that community. Study staff communicated closely with local health workers to ascertain accurate and up-to-date information on the schedule and availability of vaccination services in communities and provided tailored, personalized information on the schedule (dates, times, locations) for upcoming vaccine outreach services and local clinic-based services available to caregivers in their community. Up to three initial attempts to make contact with participants were made and additional communication continued up to 28 days of newborn life if young infants had incomplete vaccination. Participants in this arm did not receive any compensation, incentive, or reward, and CHVs were not incentivized for timely vaccinations in their communities.

#### Intervention Arm B

In intervention arm B (“CHVs and incentives”), CHVs similarly identified, documented, and reported births to central study staff and enrolled women who had recently given birth. CHVs were responsible for encouraging enrolled women in their communities to vaccinate young infants, and for providing information about the availability of local vaccination services. The timing, frequency and method of follow-up with participants was not specified and was ultimately at the discretion of the CHVs. CHVs took photos of the vaccination card and/or record with the date and location that each vaccination was received, and reported vaccinations to the central study personnel via the WhatsApp mobile phone application. The mother and the CHV were each provided a 1 Ghana cedi reward for verified, on-time vaccination with the first dose of Polio and with BCG (maximum of 2 Ghana cedis (0.50 USD) per CHV and percaregiver). Incentives were transferred to CHVs and participating caregivers via a “mobile money” phone-based banking application.

A local research staff member provided supervision to CHVs, and tracked enrollment, birth and vaccination reporting and verification in all communities. Field visits were conducted to monitor CHV activities, motivate engagement and troubleshoot challenges, and frequent communication was made with CHVs via WhatsApp to encourage participation and reporting. CHVs found not to be performing (failure to report expected number of births; failure to report vaccinations) were followed up with by local research staff and were replaced if performance problems continued.

Interventions in both treatment arms were launched on November 1, 2018; enrollment was phased out by March 31, 2019, and follow-up with enrolled particpants was completed April 30, 2019.

#### Control arm

No study-initiated activities were conducted in control communities during the intervention phase. Vaccine services continued to be available as per routine GHS health services.

### Sample selection and randomization

The GHS District Authority provided a list of all communities in Karaga and provided approval for implementing the project in the District. Communities were eligible for inclusion if they had a population size of at least 1,000 and were accessible from the main road within two hours using motorized vehicles and/or walking, during the dry season. The 15 villages with the largest populations sizes were selected from a list of all communities in the District. To avoid spillover, a minimum distance of 5 km between any two villages was imposed. Sensitization meetings with community leaders were held prior to initiation and community leaders provided permission to work in the community. Prior to randomization, research staff conducted field visits to each community to conduct a brief community survey to collect information on key characteristics considered *a priori* to be potential confounding factors. Stratified block randomization was used to achieve relative balance between communities in potential confounding factors including distance from/access to the nearest health facility; vaccine and health service delivery platform (static clinics or outreach services); population size; and distance from a main road. Random allocation to the control and the two intervention arms was assigned at the community level using max-min randomization conducted by the Principal Investigator in Switzerland [16]. All study investigators and the local research supervisor were aware of village allocation arm prior to study implementation. Following allocation in each of the ten intervention communities, the local research supervisor conducted visits with community leaders and District health workers, and hired and trained CHVs, based on the appropriate protocol for the allocation assignment. Due to the nature of the intervention, blinding of participants, investigators, and data collectors was not possible. CHV, enrolled participants, and community members were not informed of the respective research activities in other intervention arm communities, but no attempt at concealment was made. Data collectors and interviewers for the endline assessment were not engaged in program implementation and were unaware of intervention allocation, but were not blinded to the allocation. All residents of intervention communities who delivered a live-born, surviving infant during the intervention period were eligible to participate in the intervention program if the birth was identified by or reported to CHVs within the first week of life. Informed consent was obtained from all mothers or primary caregivers for program participation. Participants in each intervention arm were followed through to infant receipt of both vaccines (study endpoint) or a maximum of 28 days of newborn life.

### Vaccination coverage survey

We conducted an endline population-based survey in the 15 study communities from 30 May 2019 to 9 July 2019 to assess vaccination coverage before and after the intervention launch. Study enumerators visited every household and structure in the communities to identify all households in which a live birth had occurred in the previous year (May 2018 through May 2019) and enumerated all infants up to 12 months of age residing in the community, or born in the previous 12 months and deceased. All households with children born during the relevant time periods currently residing in the communities, for whom a caregiver provided informed consent, were eligible for inclusion in the survey. Interviewers conducted a subsequent visit to eligible households to collect informed consent and conduct a detailed interview at the household with caregivers. Interviewers returned up to two additional times if the potential participant was not available or could not be located. Vaccination history and date of birth were based on documentation in nationally standardized child health booklets and vaccination cards if available, and caregiver report if written documentation was unavailable. Photographs of child vaccination records were taken for verification. Classification into pre-intervention period or intervention period was based on the child’s documented date of birth.

Field supervisors conducted audits to ensure no households were missed during the census and and re-visited approximately 10% of eligible households identified for the survey for quality assurance purposes. Interviewers conducting the endline survey were not involved in the interventions and not aware of the treatment status of communities.

### Statistical analyses plan

The primary endpoint was on-time completion of both early vaccinations, defined as receiving at least one dose of Polio vaccine within 14 days of life and BCG vaccination within 28 days of life. Secondary endpoints were the on-time receipt of each vaccine separately; and receipt of each vaccine and both vaccines at any age. An additional secondary outcome was the proportion of births identified via the endline survey that was documented and reported by CHVs to central research staff.

#### Sample size and power

We hypothesized either intervention could result in 80% coverage of the primary endpoint, or an absolute difference of 30 percentage points relative to the 50% of children receiving both BCG and Polio vaccines within the first month after delivery from the 2014 DHS [13]. Assuming an intra-class correlation coefficient (rho) of 0.05 and 20 women per cluster, a sample size of 100 infants was required per arm to achieve a power of 90% at α of 0.05, for tests comparing each active treatment arm to control.

We anticipated with a 12-month reporting period (6 months prior to intervention and 6 months post-intervention initiation), we would identify approximately twice the number of births expected in the 6-month period, or 600 total births. To account for additional women not identified during the intervention period, who chose not to participate or moved into the community after intervention initiation, we anticipated interviewing approximately 750 women for the survey. The total number of births identified in the initial enumeration was larger than expected. To avoid exceeding the target sample size, after completing the initial population enumeration, we modified the eligibility criteria for the endline survey to limit to births in the previous 9 months. The final analysis population excluded births from April 1, 2019, onwards, which occurred after intervention enrollment had concluded (S1 Fig).

#### Analysis

The primary outcome of interest was the likelihood of young infants receiving both the first dose of the Polio and the BCG vaccine on-time. Linear regression models were used to estimate the difference in the probability of getting vaccinated on time in each of the intervention arms compared with the control arm, adjusting for baseline vaccination coverage at the community level. Base models controlled only for pre-intervention cluster-level early vaccination coverage. Adjusted models included additional controls for maternal phone ownership and phone access; mobile network coverage; time to childbirth location; location of childbirth; maternal education, household electricity, and television ownership. Primary analyses were intent-to-treat based on community-level randomized treatment assignment. Data collected as part of program implementation activities during the active intervention in intervention arm communities was not used in the primary intent-to-treat analysis. Generalized linear log-binomial regression was additionally used to estimate prevalence ratios comparing the likelihood of complete on-time early vaccination in each intervention arm relative to the control arm during the post-intervention period. In sensitivity analyses, we estimated the difference in the change in the likelihood of timely vaccination from the pre-intervention to intervention periods, in each treatment arm compared with control, among the population of births in the pre-intervention and intervention periods. Generalized linear regression models were used with control terms for the month of birth, treatment group, period (pre-intervention or intervention), an interaction term for treatment and period and other adjustment covariates as the primary model.

We also conducted an exploratory per-protocol analysis to evaluate the effect of the intervention among program participants and those successfully reached by the intervention program activities. We linked women and infants identified in the endline survey with program data, matching on the community, mother’s name, and child date of birth. In the voice call reminder arm, we defined intervention fidelity with two classifications: births documented but never successfully reached, and births documented and reached by phone at least once. In the CHV and incentives arm, intervention fidelity was defined as a birth documented by a CHV for which the caregiver agreed to participate in the program. We did not capture data on the amount, level or type of interaction of the CHV with the enrolled participants in this arm. In the per-protocol analysis, we used linear and negative binomial regression models adjusting for baseline vaccination in the intervention period only, adjusted for the same covariates previously specified. When log binomial models did not converge, we used negative binomial regressions [17].

Standard errors in all models were adjusted for correlation within clusters using robust variance estimates [18,19]. Point estimates and 95% confidence intervals were reported for all main outcomes. We did not adjust for multiple comparisons. Stata version 15 was used for analyses (StataCorp. 2017. Stata Statistical Software: Release 15. College Station, TX: StataCorp LLC.)

The trial was retrospectively registered at ClinicalTrials.gov as NCT03797950 (submission 7 November 2018, publication 9 January 2019). Publication of the registration occurred after randomization due to delays in the administrative review of the submission. This is unlikely to bias results because the endline survey and all outcome assessment data were collected after publication, and endpoints were from objective, documented records. The Ghana Health Services Ethics Review Committee approved this trial (Protocol ID 008/07/18) and Ethikkommission Nordwest-und Zentralschweiz in Switzerland determined the trial conformed to research standards for studies in Switzerland (Request 2018–00548).

## Results

### Population

**Fig 1:**
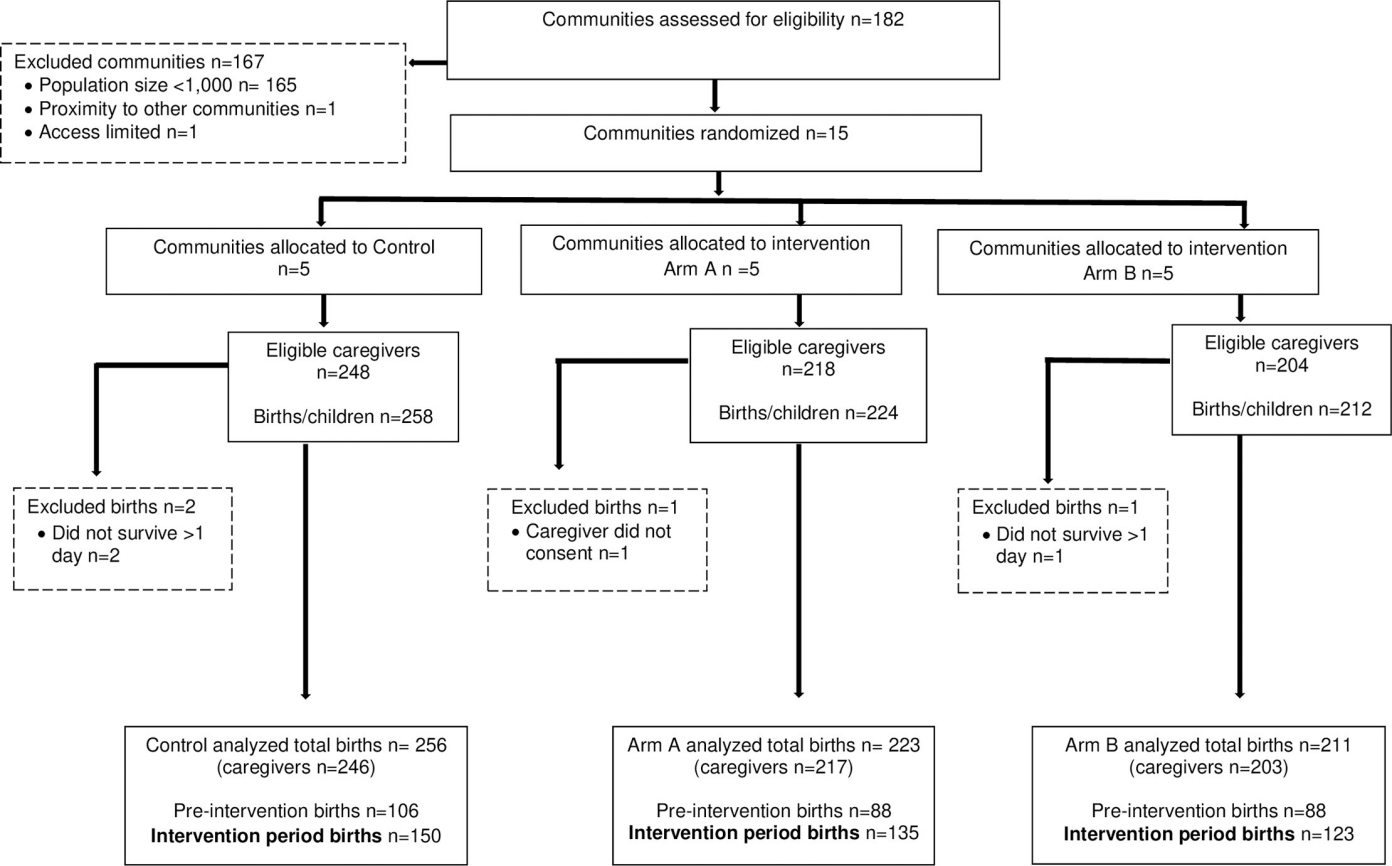
GEVaP study population flow.

Six-hundred ninety-three live births were documented in the endline survey; 690 births survived to at least one day of life and were included in the analysis; 106, 88, and 88 in the baseline period and 150, 135, and 123 during the intervention period, in the control, voice call reminders and CHV and incentive arms, respectively (Fig 1). Fig 1 describes the endline survey population flow.

Table 1 summarizes the study population characteristics by arm and time period. Approximately 80% of women surveyed had not attended any formal education. Access to a mobile phone was high, between 80–90% on average, though on average less than half of women had their own personal mobile phones and tended to share with other household or community members. Few women had access to a smartphone with the capability for internet (between approximately 4% and 20% across arms and time periods), but access was slightly higher in the incentive arm than control. Most women had received at least some antenatal care for the pregnancy, more than 95% in each arm and across time periods. Household ownership of a television and electricity, indicators of economic status, were lower in the CHV and incentive arm, on average, than control. The distribution of network coverage differed across arms, with a larger proportion of households with poor or very poor service in the control arm, and the largest proportion of households with good or very good coverage in the voice call reminder arm. Childbirth outside of a formal skilled care facility was common in all arms, but giving birth outside of a health facility, at home or in the community, was more common in both intervention arms than in the control arm. Despite balance across arms in randomization factors, communities within arms differed substantially in baseline vaccination rates, and in characteristics likely associated with neonatal vaccination.

**Table 1:** Summary of GEVaP study population.

Characteristic	Control Arm % (n)		Voice Call Reminder Arm % (n)		CHVs and Incentives Arm % (n)	
	Pre-intervention (n = 106)	Intervention (n = 150)	Pre-intervention (n = 88)	Intervention (n = 135)	Pre-intervention (n = 88)	Intervention (n = 123)
N clusters	5	5	5	5	5	5
Average cluster size (births) (min,max)	13(14,40)	30 (17,43)	18 (8,24)	27 (16,35)	18 (12,34)	25 (13,43)
*Mother and household*						
Age of mother (mean, (SD))	27.9 (6.9)	28.5 (6.0)	27.1 (85)	28.4 (5.7)	28.6 (6.9)	29.8 (7.1)
Mother’s highest level education attended^2^						
None	78.3 (83)	80.7 (121)	80.7 (71)	79.3 (107)	77.3 (68)	84.6 (104)
Primary	13.2 (14)	10.7 (16)	10.2 (9)	10.4 (14)	13.6 (12)	9.8 (12)
Secondary	8.5 (9)	8.7 (13)	9.1 (8)	10.4 (14)	9.1 (8)	5.7 (7)
Household has electricity	77.4 (82)	80.7 (121)	68.2 (60)	78.5 (106)	56.8 (50)	53.7 (66)
Household has television	41.5 (44)	48.7 (73)	37.5 (33)	36.3 (49)	30.7 (27)	28.5 (35)
Mobile phone access/ ownership (mother)						
Owns her own phone	41.5 (44)	37.3 (56)	46.6 (41)	7.4 (64)	38.6 (34)	46.3 (57)
Access to shared phone	49.0 (52)	59.3 (89)	45.5 (40)	43.7 (59)	54.6 (48)	45.5 (56)
Phone mother owns/accesses has capacity to access internet^1^						
Phone mother owns/accesses has mobile money account^1^	10.3 (9)	5.9 (8)	4.4 (3)	6.4 (7)	17.9 (10)	21.1 (16)
Network strength^1^	26.1 (24)	36.4 (52)	42.1 (32)	38.7 (46)	51.3 (39)	37.0 (40)
Very good/good	23.2 (23)	25.0 (37)	65.4 (53)	66.9 (87)	39.0 (32)	49.1 (57)
Fair	33.3 (33)	40.5 (60)	25.9 (21)	22.3 (29)	41.5 (34)	39.7 (46)
Poor or very poor	43.4 (43)	34.5 (51)	8.6 (7)	10.8 (14)	19.5 (16)	11.2 (13)
*Infant*						
Any antenatal care received	98.1 (104)	98.0 (147)	96.6 (85)	98.5 (133)	98.9 (87)	97.6 (120)
Female	43.4 (46)	44.7 (67)	51.1 (45)	46.7 (63)	47.7 (42)	41.5 (51)
Birth location^2^						
Home/ community	15.2 (16)	15.4 (23)	42.1 (37)	35.1 (47)	29.6 (26)	30.9 (38)
Hospital, Clinic or Health Center	28.6 930)	27.5 (41)	21.6 (19)	19.4 (26)	25.0(22)	28.5 (35)
Health Post or CHPS compound	56.2 (59)	57.1 (85)	36.4 (32)	45.5 (61)	45.5 (40)	40.7 (50)
Traveled 30+ min. to child birth location	46.0 (40)	46.8 (59)	50.0 (25)	43.7 (38)	50.8 (31)	62.4 (53)
Mother ever tried to vaccinate child but unable at that time	36.8 (39)	32.0 (48)	34.1 (30)	23.7 (32)	43.2 (38)	31.7 (39)
Mother ever received advice/encouragement/information about child vaccination	78.3 (83)	77.3 (116)	71.6 (63)	75.6 (102)	78.4 (69)	85.4 (105)
Vaccination card, paper of child health booklet available	97.2 (103)	96.7 (145)	95.5 (84)	98.5 (133)	95.5 (84)	97.6 (120)
Received any vaccinations in first year of life	97.2 (103)	96.7 (145)	97.7 (86)	99.3 (134)	97.7 (86)	97.6 (120)

^1^ Among those who own or have access to a phone.

^2^ Missing 1 observation each from control and reminder in post-initiation period.

^3^ Among woman who delivered in health facilities.

#### Early-infant vaccination

Almost all infants, 97% or more in all arms in both the pre-intervention and intervention periods, had received some vaccination (Table 1). The vast majority of infants, more than 95% on average in each arm, had a vaccination card or child health booklet available at the time of interview, and the proportion did not differ substantially in the pre-intervention or intervention periods or across arms. In the pre-intervention period, about a third of women in each arm (between 34–43%) had attempted to vaccinate the infant at some point but had been unable to do so. The most common reasons for unsuccessful vaccination attempts were facilities not being open or not providing services at the time of the visit; community outreach visits not occurring at scheduled or frequent enough times; vaccines not being available/stocke-outs; or health providers refusing to open vials out of fear of wastage when an insufficient number of clients were present.

In the pre-intervention period the proportion of infants vaccinated on time with BCG and the first dose of Polio differed by study arm: 44.3%, 25.0%, and 12.5% in control, voice call reminder and CHV and incentives arms, respectively (Table 2). This difference was primarily due to lower coverage of the first Polio vaccine (50.9%, 28.4%, 12.5% in control, voice call reminder and CHV and incentives arms, respectively), although timely BCG vaccination was also lower in voice call reminder and CHV and incentives arms than in control in the pre-intervention period (70.8%, 62.5% and 53.4% in control, voice call reminder and CHV and incentives arms, respectively). During the pre-intervention period the majority of infants did receive BCG, but timely coverage was less common. A smaller proportion of infants received the first dose of Polio vaccination, and receipt within the recommended 14 days of life was especially low in the CHV and incentives arm.

**Table 2:** Birth dose vaccination coverage in GEVaP study [descriptive] (N = 690).

Outcome	Control Arm % (n)		Voice Call Reminder Arm % (n)		CHVs and Incentives Arm % (n)	
Period	Pre-intervention	Intervention	Pre-intervention	Intervention	Pre-intervention	Intervention
Complete on-time vaccination^1^	44.3 (47)	48.7 (73)	25.0 (22)	37.8 (51)	12.5 (11)	54.5 (67)
On-time first dose Polio^2^	50.9 (54)	52.7 (79)	28.4 (25)	41.5 (56)	12.5 (11)	56.1 (69)
Any first dose Polio	63.2 (67)	60.7 (91)	36.4 (32)	45.2 (61)	31.8 (28)	69.9 (86)
Age at first dose Polio (days) median (IQR) ^3^	4 (1, 12)	4 (1, 10)	8 (4.5, 14)	5 (2, 10)	20 (6, 39)	6 (3, 12)
On-time BCG^4^	70.8 (75)	78.0 (117)	62.5 (55)	73.3 (99)	53.4 (47)	82.1 (101)
Any BCG	89.6 (95)	91.3 (137)	96.6 (85)	94.8 (128)	94.3 (83)	97.6 (120)
Age at BCG (days) median (IQR)^3^	12 (6, 26)	10 (3, 19)	20 (7, 36)	15 (5, 26)	26 (13, 46)	11 (5, 20)

1 Complete on-time vaccination includes BCG within 28 days of life (DOL) and at least one dose oral Polio vaccine by 14 DOL.

2 On-time BCG requires BCG vaccination within 28DOL; any refers to at any age.

3 Among those who ever received that vaccine.

4 On-time OPV0 requires at least one dose of oral Polio vaccine by 14DOL; any refers to at any age.

In the period during the GEVaP intervention, on-time vaccination coverage with the first dose of Polio and BCG was higher in all arms than during the baseline period (Table 2). In the control areas, the proportion vaccinated on time with both early vaccinations was 48.7%, a small absolute change of 4.4 percentage points from the pre-invention period. In the voice call reminder and CHV and incentives arms, 37.8% and 54.5%, respectively, of infants were vaccinated on time with both vaccines, corresponding with a crude observed increase of 12.8 percentage points in the voice call reminder arm and 42.0 percentage points in the CHV and incentives arm, from pre-intervention to intervention period. Timely vaccination coverage varied substantially across communities within and across arms in the pre- and post-initiation periods (S2 Fig).

### Program impact

**Fig 2:**
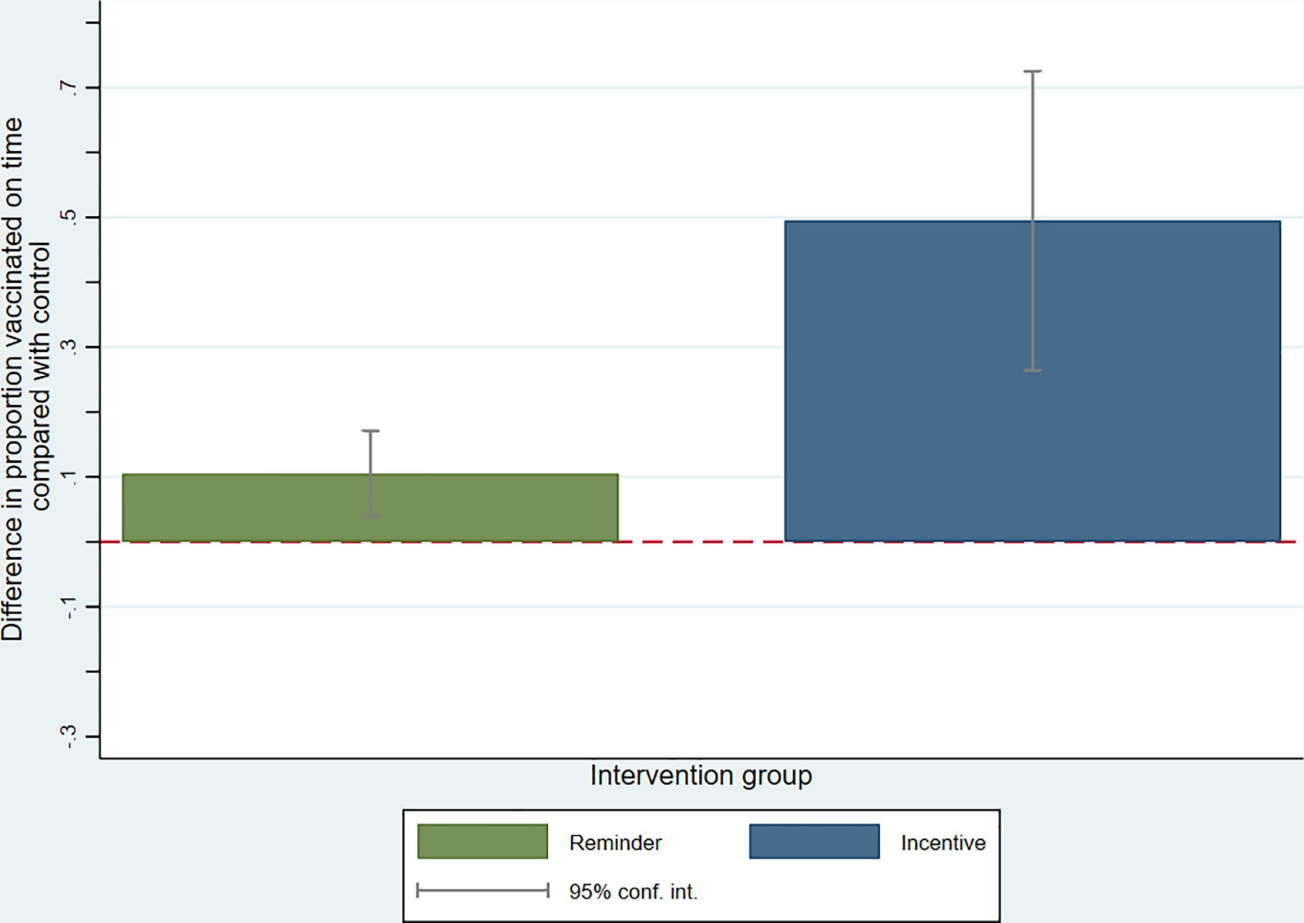
Difference in coverage of timely vaccination with first Polio and BCG vaccines with GEVaP interventions, compared with control, in post-intervention initiation period (N = 408). Difference relates to the difference in the likelihood of timely vaccination in the voice call reminder and CHV and incentives arms, compared with control arm, post-intervention initiationy. Estimates from linear regression models adjusted for baseline community-level vaccination coverage, month of birth, maternal phone ownership, network coverage, birth location, time to childbirth facility, maternal educational attainment, household electricity and TV ownership. Difference with control arm defined as β coefficient for treatment group. Variance accounts for clustering by community.

**Fig 3:**
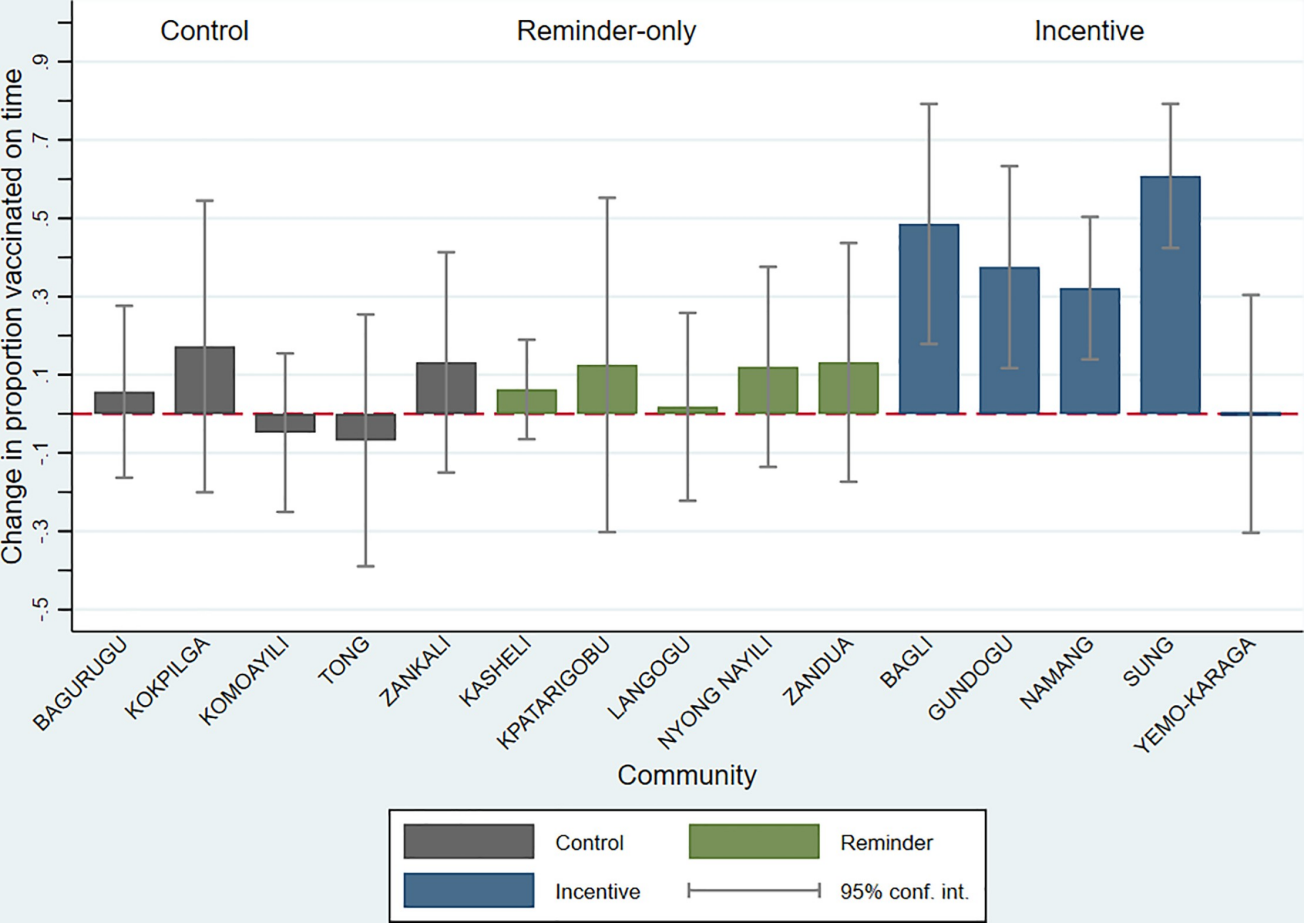
Change in proportion of young infants vaccinated on time pre-intervention and GEVaP intervention period, by community (N = 690). *Timely vaccination defined as first Polio dose and BCG by 14 and 28 days of life (DOL), respectively. Estimates from linear regression models of on-time vaccination regressed on period (pre-intervention or intervention), for each community. Change over time defined as β coefficient for period, with robust variance estimates.

In intent-to-treat analysis adjusting for baseline differences in vaccination coverage and other covariates, the proportion of infants in the voice call reminder arm who were vaccinated on time with the first dose of Polio and BCG during the intervention period was larger than in the control arm [adjusted difference in proportion (10.5 percentage point difference (95% CI: 4.0, 17.1) (Table 3) (Fig 2). The proportion of infants vaccinated on time in the CHV and incentives arm during the intervention period was 49.5 percentage points larger than in the control arm (95% CI: 26.4, 72.5). The proportion of infants with on-time vaccination with the first dose of Polio was slightly larger in the voice call reminder arm than control (10.7 percentage point difference, 95% CI: 0.30, 21.2). Timely Polio vaccination coverage was 48.8 percentage points (95% CI: 24.5, 73.1) higher in the CHV and incentives than in the control arm. Timely BCG vaccination did not differ significantly in the voice call reminder and control arms (0.16 percentage point difference, 95% CI:-1.2, 15.2), nor in the CHV and incentives and control arms (16.7 percentage point difference, 95% CI: -2.4, 35.8). The primary results did not change substantially with adjustment for other covariates, nor when estimating a difference in the change over time by intervention group as opposed to the primary post-intervention initiation estimation methods (S1 Table; S3 Fig). The magnitude of the absolute change in the proportion of infants vaccinated on time from pre-intervention to intervention period differed across communities, but CHV and incentive communities tended to have the largest and most consistent increases in coverage proportions over time (Fig 3).

**Table 3:** Effect of GEVaP intervention on timely vaccination of first Polio and BCG vaccination [Intent-to-treat] (N = 408).

	Difference in Proportion (95% CI) ^1^ (Adjusted) ^3^	Prevalence ratio (95% CI) ^2^ (Adjusted) ^3^
Complete on time birth dose vaccination ^4^		
Control	*Reference*	*Reference*
Reminder	0.105 (0.040, 0.171)	1.5 (1.1, 2.0)
Incentive	0.495 (0.264, 0.725)	4.7 (2.8, 7.8)
Timely first dose Polio		
Control	*Reference*	*Reference--*
Reminder	0.107 (0.003, 0.212)	1.5 (0.98, 2.2)
Incentive	0.488 (0.245, 0.731)	3.8 (2.0, 7.5)
Timely BCG		
Control	*Reference*	*Reference*
Reminder	0.016 (-0.121, 0.152)	1.0 (0.86, 1.2)
Incentive	0.167 (-0.024, 0.358)	1.3 (1.0, 1.6)

^1^ Difference in proportion compares the proportion vaccinated on time in each intervention arm compared with the control, in births during the intervention period, adjusted for baseline vaccination coverage, from linear regression models with robust cluster variance.

^2^ Prevalence ratios compare the proportion of young infants with timely vaccination in intervention versus control communities in births during the intervention period, adjusting for baseline differences in vaccination coverage by community, from generalized linear log-binomial regression models with robust cluster variance.

^3^ Adjusted for community baseline vaccination coverage, month of birth, location of birth, time to child birth location, maternal phone ownership and access, mobile network coverage, maternal educational attainment, household electricity and tv ownership.

^4^ Complete on-time vaccination includes at least one dose of Polio vaccine by 14 days of life (DOL) and BCG vaccine within 28DOL. Timely first dose of Polio and BCG defined as within 14 DOL 28 DOL, respectively.

### Birth reporting

Seventy-one percent (n = 96/135) of births in the voice call reminder communities and 67% (n = 82/123) in the CHV and incentives communities were identified by CHV and reported to local research staff.

### Intervention fidelity

In both arms, all caregivers approached by CHV agreed to participate in the program, and thus the population of births identified was synonymous with the enrolled program population: 71% (n = 96/135) and 67% (n = 82/123) in the voice call reminders and CHV and incentives communities, respectively, were enrolled into their respective intervention arms. Data on intervention program reach and impact among the population identified by CHVs and enrolled in the intervention program is included in Table 4. In the voice call reminder arm, less than half of the target population, 41.5%, were ever successfully reached by phone to provide a vaccination ‘nudge’ (n = 56/135). The ability to make successful contact ranged substantially across communities; only a quarter of participants were successfully reached in the lowest coverage community [26% (n = 7)] whereas 85% (n = 29) were reached successfully in the highest coverage community.

**Table 4:** Effect of participation in GEVaP intervention on timely vaccination with first dose of Polio and BCG vaccine [Per-protocol] (Intervention period N = 408).

	% (n) of target enrolled in program or reached ^2^	% (n) on-time birth doses among enrolled or reached ^6^	Difference in proportion (95% CI) (Adjusted)^7^	Prevalence ratio (95% CI) ^1^ (Adjusted)^7^
Control (N = 150)	--	48.7 (73)	*Reference*	*Reference*
Reminder (N = 135)				
Enrolled in program not reached^3^	29.6 (40)	30.0 (12)	0.055 (-0.083, 0.192)	1.3 (0.84, 2.1)
Enrolled in program reached 1+ times^4^	41.5 (56)	44.6 (25)	0.132 (0.035, 0.230)	1.7 (1.1, 2.5)
Incentive (N = 123)				
Enrolled in program^5^	66.7 (82)	61.0 (50)	0.623 (0.318, 0.927)	6.0 (3.1, 11.6)

^1^ Difference in proportion and prevalence ratio compare the likelihood of timely vaccination in the respective intervention arm with e control, among births during the intervention period. Negative binomial regression used for prevalence ratios and linear regression used to estimate difference in liklihood, adjusted for baseline vaccination coverage in the community and robust cluster variance.

^2^ Individuals in the respective intervention arm enrolled in the program and ever reached with program activities. The numerator is the size of the population enrolled or reached and the denominator is the size of the intervention arm.

^3^ Among the population identified in the endline census, comparing births in intervention communities (reminder) documented by CHVs /enrolled in the intervention, but never successfully reached by the program, to births in control communities.

^4^ Among the population identified in the endline census, comparing births in intervention communities (reminder) documented by CHVs /enrolled in the intervention that were successfully reached by the program at least once, to births in control communities.

^5^ Among the population identified in the endline census, comparing births in intervention communities (incentive) documented by CHVs /enrolled in the intervention, to births in control communities.

^6^ Individuals in the respective treatment arm, based on per-protocol classification. The numerator is the number of individuals per treatment group as the intervention was received who were vaccinated with one dose of Polio vaccine and BCG vaccine within 14 and 28 days of life, respectively, and the denominator is the number of individuals in the treatment group as the intervention was received.

^7^ Adjusted for childbirth location, time to childbirth location, phone ownership and access, mobile network coverage, maternal education, household electricity and tv ownership.

The proportion of infants vaccinated on time during the intervention period was larger among the population successfully reached at least once in the voice call reminder arm than those never reached or not identified/enrolled in that arm; 35.9% (n = 14/39) among those not enrolled in the program, 30% (n = 12/40) among those enrolled but never successfully reached and 44.6% (n = 25/56) among those successfully reached at least once (Table 4). In the CHV and incentives arm during the intervention period, 41.5% (n = 17/41) of those who were not enrolled versus 61.0% (n = 50/82) of those who participated in the program received early vaccinations on time.

### Per-protocol analysis

In the per-protocol analysis, the proportion of young infants vaccinated on time in the voice call reminder arm who were not reached with a vaccination nudge was similar to the proportion vaccinated on time in the control arm [5.5 percentage point difference (95% CI: -8.3, 19.2)]. In adjusted models, the proportion vaccinated on time among those who were successfully reached with a vaccination nudge was larger than in the control arm ([13.2 percentage point difference (95% CI: 3.5, 23.0)]. Among those with intervention fidelity, the on-time vaccination coverage was 62.3 percentage points higher (95% CI: 31.8, 92.7) in the CHV and incentives arm than in control (Table 4). Estimates of program impact were slightly larger in magnitude with adjustment (S3 Table). Vaccines with target delivery in later infancy were similar across arms (S4 Table).

## Discussion

### Implications

In this study we found that timeliness of early vaccinations in rural communities can be improved through mobile phone-based interventions delivered in the community, and are particularly effective when local CHVs implement the program and they and caregivers receive small financial incentives. An approach focusing on research staff implementation with voice calls, rather than local CHV implementation in the community, was less effective. The absolute differences in the proportion of infants vaccinated on time in both intervention arms, however, were substantial and meaningful: an additional 10.4% in the voice call reminder arm and almost fifty percent in the CHV and incentives arm.

Our study supports existing evidence that regular outreach to encourage and support vaccination can improve immunization coverage in low-resource settings, and may be especially effective when combined with household incentives. Household incentives have been associated with a nearly 7-fold increase in full vaccination coverage in settings with low coverage at baseline, and evidence from low-resource settings suggests that even small rewards to households can result in substantial increases in vaccine uptake [20,21]. Our estimates of the effect among those who were reached by the intervention were of similar magnitude: relative to the pre-intervention coverage in the same villages, on-time vaccination increased by 50% in the voice call reminder arm, and by more than 400% in the CHV and incentives arm. A study in Kenya of the effect of SMS reminders alone and with two different levels of monetary incentives also found that reminders plus cash incentives improved timely vaccination coverage, and were more effective than SMS reminders alone [22]. The incentives in our study were associated with a larger effect size. However, vaccination was relatively high in the control arm in their study, giving less opportunity to influence the absolute effect. Additionally, their approach used the same delivery model for reminders in each intervention arm (SMS), whereas in our study the incentive arm also differed from the reminder arm in the use of CHV to encourage vaccination, rather than a voice reminder, and in both arms our intervention approach used tailored reminders that also included information on where and how to access vaccinations. We also provided incentives to both CHV and to caregivers. Thus we are unable to differentiate the relative importance of the mechanism of the reminders, the incentive to the health worker and the incentive to the caregiver, in the ultimate intervention effect. Nevertheless, together, the two studies indicate that monetary incentives are more effective than reminders alone and can improve timely vaccination in both low- and high-coverage settings.

The smaller impact of the voice call reminder approach may be partially explained by the small fraction of the eligible population that was successfully reached with the intervention (42%), due to poor mobile network coverage and challenges in reaching women who didn’t have personal phones. Phone reminder interventions may be more effective in settings where mobile network coverage is more reliable and phone ownership more common. Approaches that rely on SMS or automated messaging for communicating with women and community health workers would be infeasible in similar settings due to limited literacy.

Timely vaccination coverage differed substantially across the communities at baseline, despite the relatively close geographic proximity, shared health system and public health infrastructure and similar demographic and ethnic characteristics of communities. Communities differed in the relative geographic proximity to health services and the frequency with which these services were made available to them, which may have influenced both baseline coverage and the ability of the demand-side intervention to improve coverage in some communities. Variation across communities is also partially explained by differences in network coverage and the ability to access and reach participants in the voice call reminder arm, and the level of engagement of volunteers and volunteer program uptake in the CHV and incentives arm. Additionally, communities with low levels of coverage at baseline had more opportunity for an absolute increase in coverage. Future implementation research should evaluate factors that influence intervention uptake and effect at the community level.

Future research to elucidate the factors driving vaccination practices in these and similar settings will be essential to designing effective interventions. This intervention focused on increasing the demand for vaccination among caregivers, but timely vaccination also requires that vaccines are available and accessible. Timely vaccination was dependent on the timing and frequency of outreach services in communities and the availability of services at local health facilities. Yet outreach services were available at most once a month; not frequent enough for many infants to receive early vaccines within the recommended window, and services were frequently canceled or rescheduled for other campaigns, further limiting timely access. Participants also reported that health workers often would not open vials because they feared wasting vaccine doses. Such system-level bottlenecks to access must also be addressed for demand-side interventions to have impact.

Our research specifically evaluated the impact of interventions on neonatal vaccines, but a similar model could be adapted to support engagement with vaccination and child health preventive services throughout infancy and childhood. We did not observe differences in coverage of vaccines delivered later in infancy; it is likely that to influence behavior an incentive structure that continues to provide rewards over time would be required to influence longer-term engagement with the health system. Local research staff conducted ongoing supervision and close quality assurance during the study period and found that CHV motivation was often a challenge. Supervision systems and appropriate remuneration for CHVs will be essential to maintain impact in similar programs. To be effective, the incentive must be large enough to initiate behavior-change, motivate action and offset any perceived costs or barriers, for both the CHVs and thecaregivers. Thus policy-makers must consider the cost of not only the incentives, but of developing and maintaining a support and supervision structure that will encourage ongoing engagement and performance.

CHV’s documented the majority of births in their communities within the first week of life, and in the absence of the program, historically, approximately 40% of births are not registered with local authorities and have not been issued a birth certificate in the first year of life [14]. Similar simple community-initiated early birth identification and reporting systems could be integrated into the local civil registration systems to improve timeliness and completeness of birth registration.

### Strengths

We conducted a pragmatic trial embedded within the local public health service delivery structure. Our strategy required limited resources and engaged local community members previously trained in health promotion, to engage in activities to increase demand for and utilization of vaccination services that were already available and to help connect potential clients with available services. The intervention approach was widely acceptable to community members and caregivers, and required limited financial resources or health system infrastructure.

### Limitations

Vaccination coverage was not perfectly balanced across arms in the pre-intervention period, due to the small number of communities and large variability in community coverage. However, our assessment of outcomes pre-intervention and post-intervention initiation allowed adjustment for these differences in the analyses.

The external validity of our results and the ability to explore factors associated with vaccination practices and program impact were limited by the small number of communities. On average, the study population was rural, had low educational attainment and literacy, and commonly delivered children outside of a health facility. Despite widespread access to cell phones and use of mobile financial transfer applications, few woman had personal phones and mobile network coverage was often poor. The population was served by a community level health system infrastructure through which vaccination services were available, and overall vaccination coverage was relatively high, but childhood vaccinations were frequently received late. Although the barriers to timely vaccination differ in different settings, and intervention effect will vary based on both individual and community-level factors, the results should be widely generalizable to similar populations in other resource-limited settings.

To test the potential feasibility of the intervention approach, we focused on a relatively short period and a limited number of health service use outcomes. Thus we are unable to determine whether a similar approach could be used to improve coverage of additional preventive health services and later childhood vaccinations. Some studies with longer periods of follow-up indicate the effect of incentives may diminish over time, and may not be effective at sustaining long-term health system engagement. We are unable to differentiate the respective contributions of the different components of each intervention approach on the outcomes observed, and thus can’t differentiate the influence of the incentive for CHVs, incentive for caregiver and method and intensity of interaction with caregivers. The approaches in the two active intervention arms included multiple components and differed in multiple ways; arm A focused on research study staff as the key implementers; used voice call reminders at structured intensity and time points, rather than community-level visits or interactions; and did not include incentives for vaccination for caregivers or for CHVs. Arm B focused on using CHVa as key implementers; allowed freedom in the mode and intensity of interactions with caregivers; and provided incentives to both CHV and caregivers. We evaluated the effect of only a single, small level of incentive for CHVs and caregiversabout the cost of purchasing breakfast from a local street vendor. Future research should evaluate the optimal incentive level and structure to motivate health workers and caregiversn and to sufficiently sustain motivation. Incentive structures that vary the level or timing of the payment based on the level of effort or perceived barriers required, or provide incremental payouts for interim and final milestones may impact health worker and client motivation over time. Optimal incentive costs will need to balance the intervention impact with financial feasibility, given available resources and budgetary constraints in different settings.

Due to the limited literacy in the communities, and the variability in service availability schedules, personal voice calls were made rather than automated voice messages or SMS reminders, which would be timely and expensive to scale. Automated technologies appropriate for low-literacy settings could improve the cost-effectiveness and ability to scale, but would require that services are consistently available or would require a mechanism to link updated information about service schedules with clients in specific settings.

### Conclusion

Community-based outreach and small financial incentives to community volunteers and caregivers improved the timeliness of early vaccination in rural communities. Mobile phone voice call-based interventions are likely to miss a substantial proportion of the target population in settings where network coverage and phone ownership remain limited, yet such approaches may be effective in well-connected populations. Further research to test the effect of similar approaches on improving coverage of other infant and child health vaccines and preventive services and to determine the appropriate incentive and supervision structure is needed.

## Supporting information

S1 Fig S1 Fig GEVaP Study and survey timeline. (DOCX)

S2 Fig Coverage of timely vaccination with first Polio and BCG vaccines in GEVaP communities pre-intervention and GEVaP intervention period. (DOCX)

S3 Fig Change in coverage of timely vaccination with first Polio and BCG vaccines pre-intervention and GEVaP intervention period. (DOCX)

S1 Table Effect of GEVaP intervention of timely vaccination of first polio and BCG vaccination [Intent-to-treat] [Intervention period, N = 408] (Unadjusted analysis). (DOCX)

S2 Table Effect of GEVaP intervention of timely vaccination of first polio and BCG vaccination [Intent-to-treat] [Change from pre-intervention to intervention period, N = 690]. (DOCX)

S3 Table Effect of participation in GEAVaP intervention on timely vaccination with first dose of Polio and BCG vaccine [Per-protocol] (Intervention period N = 408) (Unadjusted analysis). (DOCX)

S4 Table On-time coverage of infant vaccinations in GEVaP intervention and control communities (N = 690). (DOCX)

S1 File Consort checklist. (DOCX)

S2 File TIDieR checklist. (DOCX)

S3 File Study protocol. (PDF)

## Abbreviations

BCG: Bacillus Calmette–Guérin
CHV: Community Health Volunteers
EKNZ: Ethikkommission Nordwest und Zentralschweiz
GHS: Ghana Health Service
IPA: Innovations for Poverty Action
SMS: Short message service
Swiss TPH: Swiss Tropical and Public Health Institute
