# Supplementary figures and images for: Mobile nudges and financial incentives to improve coverage of timely neonatal vaccination in rural areas (GEVaP trial): A 3-armed cluster randomized controlled trial in Northern Ghana
Source: PLoS One. 2021 May 19;16(5):e0247485. doi: 10.1371/journal.pone.0247485 (PMC8133473; doi:10.1371/journal.pone.0247485)

**S1 Figure GEVaP Study and survey timeline**


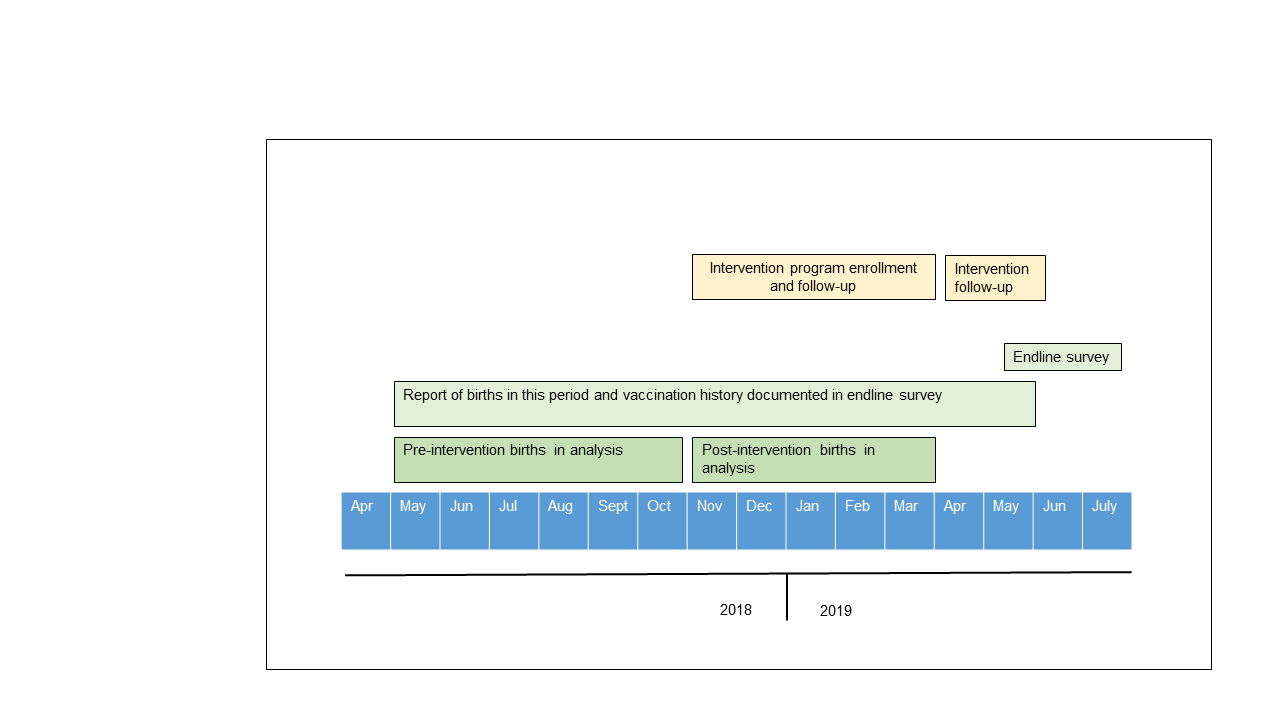

Supplement: S1 Fig — (DOCX) [file pone.0247485.s001.docx]
