# Supplementary material for: Mobile nudges and financial incentives to improve coverage of timely neonatal vaccination in rural areas (GEVaP trial): A 3-armed cluster randomized controlled trial in Northern Ghana
Source: PLoS One. 2021 May 19;16(5):e0247485. doi: 10.1371/journal.pone.0247485 (PMC8133473; doi:10.1371/journal.pone.0247485)
Supplement: S2 Fig — (DOCX) [file pone.0247485.s002.docx]

**S2 Figure. Coverage of timely vaccination with first Polio and BCG vaccines in GEVaP communities pre-intervention and intervention period**

*Timely vaccination defined as first Polio dose by 14 days of life and BCG by 28 days of life
