# Supplementary material for: Mobile nudges and financial incentives to improve coverage of timely neonatal vaccination in rural areas (GEVaP trial): A 3-armed cluster randomized controlled trial in Northern Ghana
Source: PLoS One. 2021 May 19;16(5):e0247485. doi: 10.1371/journal.pone.0247485 (PMC8133473; doi:10.1371/journal.pone.0247485)
Supplement: S3 Fig — (DOCX) [file pone.0247485.s003.docx]

**
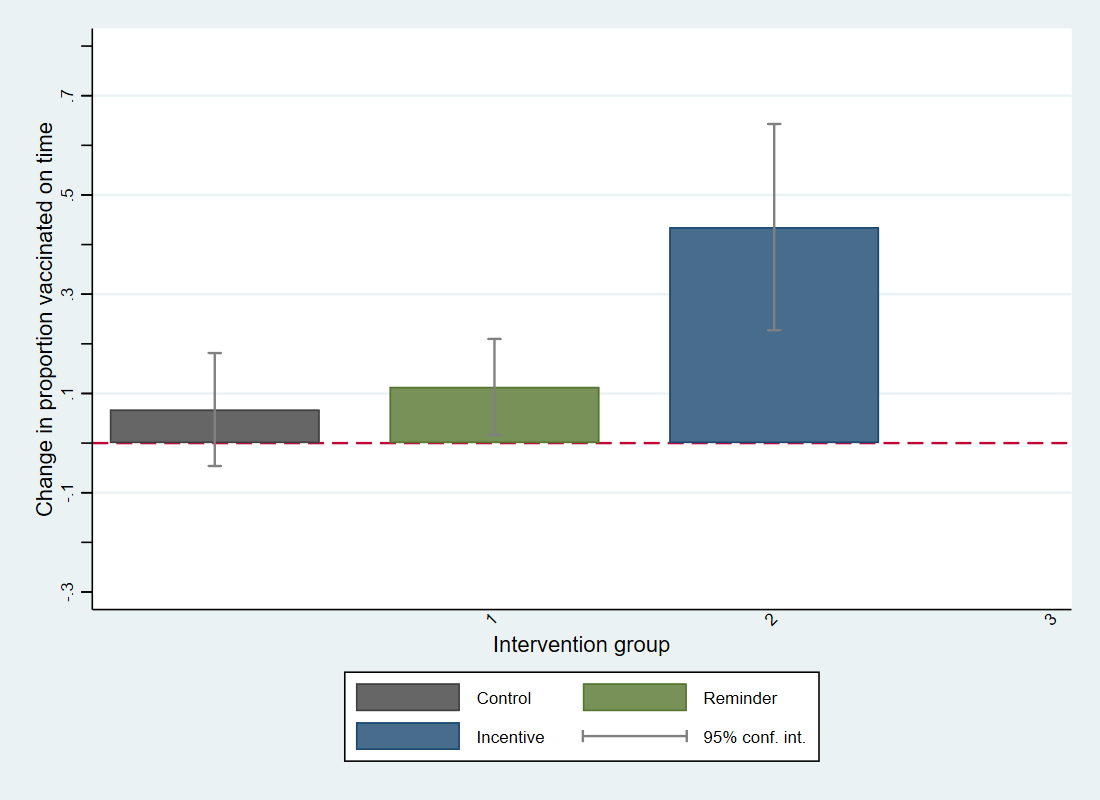
S3 Figure. Change in coverage of timely vaccination with first Polio and BCG vaccines pre-intervention and GEVaP intervention period**

*Timely vaccination defined as first Polio dose by 14 days of life and BCG by 28 days of life. Estimates from adjusted linear regression models of on-time treatment regressed on period (pre-intervention versus intervention), treatment arm, an interaction term for period and treatment arm, community, month of birth, maternal phone ownership and access, mobile network coverage, birth location, time to childbirth facility, maternal educational attainment, household electricity and TV ownership. Change over time defined as sum of β coefficients for period and period-by-treatment interaction term. Variance accounts for clustering by community.
