# Supplementary material for: Mobile nudges and financial incentives to improve coverage of timely neonatal vaccination in rural areas (GEVaP trial): A 3-armed cluster randomized controlled trial in Northern Ghana
Source: PLoS One. 2021 May 19;16(5):e0247485. doi: 10.1371/journal.pone.0247485 (PMC8133473; doi:10.1371/journal.pone.0247485)
Supplement: S1 Table — (DOCX) [file pone.0247485.s004.docx]

**S1 Table. Effect of GEVaP intervention of timely vaccination of first polio and BCG vaccination [Intent-to-treat] (Intervention period N = 408) (Unadjusted analysis)**

|  | Difference in proportion (95% CI) ^2^ | Prevalence ratio  (95% CI) ^1^ |
| --- | --- | --- |
| Complete on time birth dose vaccination  Control  Reminder  Incentive l | *Reference*  **0.087 (0.006, 0.167)**  **0.474 (0.245, 0.702)** | *Reference*  1.4 (0.99, 1.9)  **4.1 (2.4, 6.0)** |
| Timely first dose polio  Control  Reminder  Incentive | *Reference*  **0.101 (0.002, 0.201)**  **0.475 (0.242, 0.708)** | *Reference*  1.4 (0.96, 2.1)  **3.6 (1.8, 6.9)** |
| Timely BCG  Control  Reminder  Incentive | *Reference*  -0.027 (-0.150, 0.97)  0.131 (-0.053, 0.315) | *Reference*  0.96 (0.82, 1.1)  1.2 (0.96, 1.5) |

Complete on-time vaccination includes at least one dose of polio vaccine by 14 days of life and BCG vaccine within 28 days of life, either documented or reported. Timely first dose of polio defined as within 14 day of life and timely BCG defined as within 28 days of life, either documented or reported.

^1^ Prevalence ratios compare the proportion of young infants with timely vaccination in intervention communities versus control communities in births during the intervention period, adjusting for baseline differences in vaccination coverage by community, from generalized linear model log-binomial regression models with robust cluster variance estimates.

^2^ Difference in the proportion compares the proportion of young infants vaccinated on time in each intervention arm compared with the control arm, during the intervention period, adjusting for baseline coverage, from linear regression models with robust cluster variance estimates.

Bold indicates statistical significance at α level 5%
