# Supplementary material for: Mobile nudges and financial incentives to improve coverage of timely neonatal vaccination in rural areas (GEVaP trial): A 3-armed cluster randomized controlled trial in Northern Ghana
Source: PLoS One. 2021 May 19;16(5):e0247485. doi: 10.1371/journal.pone.0247485 (PMC8133473; doi:10.1371/journal.pone.0247485)
Supplement: S2 Table — (DOCX) [file pone.0247485.s005.docx]

**S2 Table. Effect of GEVaP intervention of timely vaccination of first polio and BCG vaccination [Intent-to-treat] [Change from pre-intervention to intervention period, N=690]**

| Pre-intervention period and intervention period N= 690 | | |
| --- | --- | --- |
|  | Percentage point difference in change (95 % CI)^1^ | |
|  | Base model ^2^ | Adjusted ^3^ |
| Complete on time birth dose vaccination  Control period charge  Reminder  Incentive l | -0.011 (-0.375, 0.352)  0.049 (-0.047, 0.144)  **0.365 (0.167, 0.564)** | -0.009 (-0.374, 0.357)  0.058 (-0.040, 0.154)  **0.363 (0.172, 0.549)** |
| Timely first dose polio  Control period change  Reminder  Incentive | -0.018 (-0.388, 0.351)  0.072 (-0.044, 0.188)  **0.399 (0.205, 0.592)** | -0.236 (-0.353, -0.120)  0.081 (-0.400, 0.341)  **0.395 (0.201, 0.589)** |
| Timely BCG  Control period change  Reminder  Incentive | 0.005 (-0.213, 0.223)  -0.004 (-0.160, 0.152)  **0.228 (0.021, 0.435)** | 0.000 (-0.262, 0.263)  -0.003 (-0.137, 0.131)  **0.237 (0.037, 0.437)** |

Complete on-time vaccination includes at least one dose of polio vaccine by 14 days of life and BCG vaccine within 28 days of life, either documented or reported. Timely first dose of polio defined as within 14 day of life and timely BCG defined as within 28 days of life, either documented or reported.

^1^ Effect estimates are from generalized linear regression models to estimate the difference between each intervention arm and the control arm (reference) in the change in coverage from pre-intervention to intervention period. The control-arm effect estimate is the difference in the control arm from pre-intervention to intervention period. The effect estimate relates to the percentage point difference in the change in the proportion of young infants vaccinated on time from pre-intervention to intervention period, comparing each intervention arm to the control arm, and is parameterized by the $\beta$coefficient of the treatment-arm-by-period interaction term. Population includes births in pre-intervention and intervention periods.

^2^ Base and adjusted models adjusted for community and month of birth, with variance adjustments for clustering by community.

^3^ Adjusted model additionally adjusted for location of birth, time to birth location, maternal phone ownership and access, mobile network coverage, maternal educational attainment, household electricity and tv ownership.

Bold indicates statistical significance at α level 5%
