# Supplementary material for: Mobile nudges and financial incentives to improve coverage of timely neonatal vaccination in rural areas (GEVaP trial): A 3-armed cluster randomized controlled trial in Northern Ghana
Source: PLoS One. 2021 May 19;16(5):e0247485. doi: 10.1371/journal.pone.0247485 (PMC8133473; doi:10.1371/journal.pone.0247485)
Supplement: S3 Table — (DOCX) [file pone.0247485.s006.docx]

**S3 Table. Effect of participation in GEAVaP intervention on timely vaccination with first dose of Polio and BCG vaccine [Per-protocol] (Intervention period N= 408) (Unadjusted analysis)**

|  | % (n) of target enrolled in program or reached^1^ | % (n) on-time birth doses among enrolled or reached ^2^ | Difference in proportion (95% CI ) ^4^ | Prevalence ratio  (95% CI) ^3^ |
| --- | --- | --- | --- | --- |
| Control (N= 150)  Reminder (N= 135)  Enrolled in program not reached^5^  Enrolled in program reached 1+ times^6^  Incentive (N= 123)  Enrolled in program^7^ | --  29.6 (40)  41.5 (56)  66.7 (82) | 48.7 (73)  30.0 (12)  44.6 (25)  61.0 (50) | *Reference*  0.038 (-0.131, 0.208)  **0.095 (0.017, 0.174)**  **0.582 (0.323, 0.840)** | *Reference*  1.2 (0.64, 2.3)  1.4 (0.98, 2.1)  **4.9** **(2.4, 9.9)** |

Bold indicates statistically significant at $\alpha$ level 5%

^1^n and % relate to the number of individuals and proportion of individuals in the respective intervention arm who were enrolled in the program and ever reached by the program activities. The numerator is the size of the population enrolled in the program or reached by the program in the intervention time period and the denominator is the size of the intervention arm in the intervention time period.

^2^ Prevalence ratio and difference in proportion compares proportion of young infants with complete early vaccination in the respective intervention communities with the proportion in the control communities, among births during the intervention period, adjusted for baseline. Negative binomial regression model adjusted for baseline vaccination coverage in the community, used for prevalence ratios and linear regression used to estimate difference in proportion, with robust cluster variance by community.

^3^ Difference in proportion compares proportion of young infants with complete early vaccination in the respective intervention communities with the proportion in the control communities, among births during the intervention period, adjusted for baseline. Negative binomial regression model adjusted for baseline vaccination coverage in the community, used for prevalence ratios and linear regression used to estimate difference in proportion, with robust cluster variance by community.

^4^ Among the population of women identified in the endline census, comparing births in intervention communities (reminder) that were documented by CHVs /enrolled in the intervention, but were never successfully reached by the GEVaP program, to births in control communities

^5^ Among the population of women identified in the endline census, comparing births in intervention communities (reminder) that were documented by CHVs /enrolled in the intervention, and were successfully reached by the GEVaP program at least once, to births in control communities

^6^ Among the population of women identified in the endline census, comparing births in intervention communities (incentive)) that were documented by CHVs /enrolled in the intervention, to births in control communities

^7^ Relates to the number and proportion of individuals in the respective treatment arm, based on per-protocol (as-treated) classification, who were vaccinated on time, The numerator is the number of individuals per treatment group as the intervention was received who were vaccinated with one dose of polio vaccine within 14 days of life and BCG vaccine within 28 days, and the numerator in the number of individuals in the treatment group as the intervention was received.
