# Supplementary material for: Mobile nudges and financial incentives to improve coverage of timely neonatal vaccination in rural areas (GEVaP trial): A 3-armed cluster randomized controlled trial in Northern Ghana
Source: PLoS One. 2021 May 19;16(5):e0247485. doi: 10.1371/journal.pone.0247485 (PMC8133473; doi:10.1371/journal.pone.0247485)
Supplement: S4 Table — (DOCX) [file pone.0247485.s007.docx]

**S4 Table. On-time coverage of infant vaccinations in GEVaP intervention and control communities (N= 690)**

|  | Control Arm % (n=) | | Reminder-only Arm % (n) | | Incentive Arm % (n) | |
| --- | --- | --- | --- | --- | --- | --- |
| Period | Pre-intervention | Intervention | Pre-intervention | Intervention | Pre-intervention | Intervention |
| 6 week vaccines  OPV 1  DPT/Hap B/Hib 1  Pneumococcal 1  Rotavirus 1  10 week vaccines  OPV 2  DPT/Heb B/ Hib 2  Pneumococcal 2  Rotavirus 2 | 62.3 (66)  50.9 (54)  54.7 (58)  66.0 (70)  31.1 (33)  27.1 (28)  27.4 (29)  33.0 (35) | 68.7 (103)  63.3 (95)  68.7 (103)  68.0 (102)  37.2 (55)  35.7 (51)  37.2 (55)  37.8 (56) | 51.1 (45)  42.1 (37)  45.5 (40)  46.6 (41)  26.1 (23)  23.8 (20)  26.1 (23)  26.1 (23) | 48.9 (66)  50.4 (68)  49.6 (67)  49.6 (67)  24.1 (32)  25.2 (33)  24.8 (33)  24.1 (32) | 38.6 (34)  23.9 (21)  31.8 (28)  38.6 (34)  19.3 (17)  13.1 (11)  14.8 (13)  19.3 (17) | 49.6 (61)  49.6 (61)  49.6 (61)  50.4 (62)  13.9 (17)  16.0 (19)  13.9 (17)  15.8 (18) |

On time coverage defined as receipt of vaccination within 2 weeks of national vaccine schedule recommendation (6 week vaccines by 8 weeks of life; 10 week vaccines by 3 months of life). Denominator for coverage of each vaccine includes all infants old enough to have received the vaccine at the time of the survey.
