# Supplementary material for: Mobile nudges and financial incentives to improve coverage of timely neonatal vaccination in rural areas (GEVaP trial): A 3-armed cluster randomized controlled trial in Northern Ghana
Source: PLoS One. 2021 May 19;16(5):e0247485. doi: 10.1371/journal.pone.0247485 (PMC8133473; doi:10.1371/journal.pone.0247485)
Supplement: S3 File — (PDF) [file pone.0247485.s010.pdf]

Study Protocol:

Mobile Nudges to Increase Early Vaccination Coverage in Rural Areas – A Pilot Investigation in Ghana's Northern Region

Project summary

Despite major progress made in vaccination coverage overall, timeliness of vaccines remains a key concern in many settings. At the same time, access to mobile phones has increased rapidly, offering new opportunities to track and deliver health services. The main idea of this research project is to use these newly available mobile phone networks to simultaneously address two of the biggest bottlenecks in vaccine delivery: timely registration of births, and lack of maternal effort to get essential vaccines. To increase registration, we will train volunteers selected by each community to report new births via SMS to a central registration system, and send small monetary rewards via mobile phone to volunteers for this reporting. To increase vaccination coverage, we will send reminder messages directly to mothers, and will also test small monetary rewards as an incentive to complete recommended vaccinations. The vaccination encouragement designs will be tested through a small randomized controlled study in 15 selected villages in Ghana's Northern region. The primary outcome variable for the pilot study will be the percentage of children with both completed BCG and initiated Polio vaccinations within the first four weeks (28 days) of their life.

General information

**Protocol title:**	Mobile Nudges to Increase Early Vaccination Coverage in Rural Areas – A Pilot Investigation in Ghana's Northern Region
**Funding**	Bill and Melinda Gates Foundation (Grand Challenges Exploration Grant Round 20)
**Sponsor/Principal Investigator (PI):**	Prof. Günther Fink
**PI Contact Information**	Swiss Tropical and Public Health Institute, Socinstrasse 57, 4051 Basel, Switzerland
**Local Contact Information**	Amadu Salifu, Innovations for Poverty Action, HNo B58, Old Ghanem yard, Zogbeli, Tamale, Ghana.

Rationale & background information

Despite major progress made in vaccination coverage overall, timeliness of vaccines remains a key concern in many settings [1, 2]. In Ghana, 95% of children receive BCG vaccination within the first two years of life [3], but only about half of these children receive the vaccine within the first 30 days after delivery, and more than 25% receive the vaccine more than 6 months after birth [3]. Early vaccination coverage is particularly low in settings where a large share of women deliver at home, as it is the case in Ghana's Northern region, where only a minority of women seek out facilities for delivery [3]. According to the latest estimates, less than 50% of children in Ghana's Northern region receive Bacillus Calmette–Guérin (BCG) vaccines within the first three months of their life, and less than 40% get polio vaccination, exposing a large number of infants to these diseases [2].

Recent research conducted in this region shows that the large majority of households have access to mobile phones now even in the most rural areas [4]. The main idea of the proposed project is to use these mobile phone networks to simultaneously address two of the biggest bottlenecks in vaccine delivery: timely registration of births, and lack of maternal effort to get essential vaccines. Evidence from India

suggests that even small rewards can often result in substantial increases in vaccine uptake [5] – we will test this hypothesis using mobile networks in rural areas of Northern Ghana.

Study goals and objectives

The main objective of this study is to assess the extent to which mobile-phone based reminder or reward systems can increase early vaccination coverage. We will assess two specific interventions through the pilot study:

1) an *automated call and text system* which will first call mothers to highlight the importance of early vaccinations, and then send further text reminders (nudges) to mothers 3, 7 and 10 days after birth encouraging them to get their newborn vaccinated.

2) a *community volunteer-based system* that will provide small mobile credit rewards to both community volunteers and mothers for completing the early vaccinations within the first two weeks of newborn life.

Research Hypotheses (H1-3)

H1: Nudging mothers through voice and text messages will increase early vaccination coverage.

H2: Nudging mothers through community volunteers and small rewards will increase early vaccination coverage.

Given that birth registration remains low in many parts of Ghana, we will also test a new community-based reporting model, under which volunteers appointed by the community will receive small rewards for reporting births in their communities. We will test the extent to which such a program improves birth reporting and registration:

H3: Providing small mobile-phone based incentives to volunteers selected by communities will result in accurate and timely reporting of births in rural areas.

Literature Review

A large number of studies have explored the utility of text reminders for the provision and utilization of health services. A recent review of mobile telephone text messaging (short message service, SMS) identified 60 studies assessing the impact of mobile phone based reminders on health service utilization [6]. Most studies in the current literature focus on adherence to medication, such as malaria [4], HIV [7] and management of chronic illness, such as diabetes and hypertension [8]. Systematic reviews for both HIV and long-term self-managed care suggest moderate size, but generally statistically significant increases in adherence to treatment [6, 7]. Mobile text messages have also been increasingly used to increase attendance of health care appointments: the latest systematic review suggests that text reminders can increase attendance of scheduled appointments significantly and are likely a cost-effective tool in this setting [9]. Evidence is much more limited on preventive behavior; a 2012 Cochrane review identified only one moderate quality study focusing on vitamin C intake, and a second high quality study focusing on smoking cessation [10]. In a more recent review focusing directly on vaccination, six randomized controlled trials using text reminders were identified; all six of these trials were conducted in high income settings, and focused mostly on older populations [11]. To our knowledge, there is no study that has looked at the effectiveness of mobile text reminders for increasing early vaccination coverage in a low income setting.

In terms of alternative interventions to improve vaccination coverage in low income settings, regular immunization outreach has been identified to be most effective in improving full immunization coverage, especially if combined with household incentives (the last Cochrane review finds a risk ratio of 6.66 for full vaccination coverage, even though this evidence was classified as low-certainty) [12].

Given this background, we believe that the proposed interventions have high potential to improve early vaccination coverage, and hope that the proposed pilot investigation will provide first evidence in support of this hypothesis.

Study Design Overview

The study will be designed as an open label cluster-randomized controlled study with three arms: a control arm, a reminder-only arm, and a reward arm. During the intervention phase community volunteers will be appointed to register all births in 10 randomly selected treatment communities. In each active treatment arm, an estimated 100 women across five villages will be enrolled in a proactive program. In intervention group A, participating women will receive reminders about vaccinations recommended at birth via mobile phones. In intervention group B, participating women will receive encouragement from a community-appointed volunteer to complete vaccinations recommended as well as a small cash reward for completing the recommended vaccinations. An estimated total of 200 women in the 10 treatment villages will receive one of the two study-initiated program interventions. No volunteer will be appointed to register births in the 5 control-arm villages and no woman in the 5 control-arm villages will be actively enrolled nor receive a pro-active program intervention during the intervention phase.

After the intervention phase is complete, an endline population-based household survey will be conducted in the 15 study villages to evaluate the effect of the intervention programs. The survey will enroll all consenting woman who have delivered a live-born baby in the last 12 months and reside in one of the 15 communities in the three study arms (10 intervention communities and 5 control communities). Based on a crude birth rate of 40 and an average village size of 100-1500 people, we estimate enrolling approximately 600-750 women in the endline survey. The endline survey will be used to compare the coverage of recommended early newborn vaccinations, and the proportion of births accurately and completely registered in each arm.

Methodology

To assess the feasibility and effectiveness of the proposed early vaccination support platforms, we will conduct a pilot study in 15 rural communities in Northern Ghana. The 15 communities will be divided into 3 groups: 1) a **reminder-only intervention group** (Intervention Group A, 5 communities), 2) an **incentivized reward group** (Intervention Group B, 5 communities); and a **control group** (5 communities). Approximately 200 mothers (100 per treatment arm) will be enrolled in intervention programs across the two treatment groups.

Community Volunteer Appointment and Birth Registration

In all of the 10 intervention arm communities, we will implement sensitization meetings at the beginning of the study to explain the purpose and objective of the proposed work. We will then ask the community to appoint a local birth registration volunteer. Minimum qualifications for the local birth registration volunteer include the following: female; owns and has daily access to a smart phone with camera capability and the knowledge and ability to take photos and send SMS messages; is a member of the local community and in good standing with the community; speaks the local language fluently and has at least a minimum working knowledge of English and Dagbani; can read and write (local language fluency and working knowledge of English).

Candidate volunteers will be informed of the expectations of participating in the project, the project duration (6 months), the expected time commitment per week (approximately one hour per week in the incentive program areas and 30 minutes per week in the reminder-only program areas). They will then be invited to accept or decline the appointment as a birth registration volunteer. Birth registration volunteers who accept the appointment will undergo a standardized training to further inform them of the procedures and expectations of their role, and to provide practical advice on successfully engaging in their role.

Community volunteers will be expected to document and report all births in their communities to a central phone hotline which will be managed by study personnel. To motivate volunteers to report all births in their communities, and to compensate them for their efforts, they will be given a 2 cedis reward (USD 0.50) for each birth reported during the active study duration (6 months).

Intervention arm A (***"reminders only")*** focuses on automated voice and text messages sent to mothers. In this treatment arm, community volunteers will register all births in the community as described above.

All women who live in this community and have given birth to a live-born newborn in the previous week are eligible for enrollment. As soon as possible after each birth, volunteers will contact the mother to briefly introduce the study and ask if they are interested in receiving vaccination-related information via voice messages and SMS on their phones. If the woman is interested, volunteers will describe the study purpose and procedures in more detail, answer any questions the woman has about participation, and guide the woman through the informed consent process. If mothers sign the written informed consent document (see *Informed Consent Reminder Intervention*), volunteers will ask the woman to provide a phone number of their choice at which they would like to be contacted to receive vaccine-related information and reminders. The volunteers will send the phone number provided by enrolled woman, together with a copy of the signed consent form, to a central study system.

An initial (automated) voice call will be made to all enrolled mothers to congratulate the mother on the birth and to highlight the importance of early vaccinations for their newborns. The message will also recommend that the mother take the newborn to get the BCG and Polio0 vaccine as soon as possible after birth if the newborn has not yet received these vaccines, ideally within two weeks after birth (as per Ghana national recommendations on immunization schedule). Additional reminder text messages will be sent to the phone number provided to the mother at 3 days, 7 days and 10 days of newborn life (as appropriate), reminding the woman to complete these vaccinations for her child. The text reminders will be short messages that reiterate the importance of early newborn vaccination for BCG and polio and will encourage woman to seek these vaccines for their newborns within the first two weeks of life if the newborn has not yet received them.

All mobile communications will include a note stating that if the woman prefers to stop receiving additional reminders or information she can flash (briefly call) the study phone line and they will call her back at no cost to her, so that she can request to stop receiving messages. She will also be informed that she can send a simple "STOP" text message to the same mobile number which sent the reminder (or voice message). Messages will continue to be sent to the woman through approximately the first two weeks of newborn life. The duration of follow-up with enrolled woman will be two weeks after enrollment in the study. Community birth registration volunteers will not contact enrolled participants in this arm (or women who meet eligibility criteria but chose not to be enrolled) after the completion of the birth registration and informed consent procedure is completed.

Enrolled participants in this arm will not receive any monetary compensation, incentive or reward for participation.

Intervention arm B ("incentives") relies more directly on community volunteers. In the incentives arm, community volunteers will similarly register all births in their community during the intervention phase. The volunteers will receive a 2 cedis phone credit reward for each birth registered in their community.

All women who reside in the community and have given birth to a live-born newborn in the previous week are eligible for enrollment. Volunteers will contact mothers as soon as possible after birth and will introduce the study purpose and procedures and ask if the woman may be interested in participating. If the woman is interested the volunteer will guide them through the informed consent process and will describe in more detail the study purpose and procedures and answer any questions the woman may have about participation. If the woman then agrees to participate and signs the written informed consent document they will be enrolled in the study (see *Informed Consent Incentives*). No incentive will be provided to the volunteer or to the mother on the grounds of study enrollment.

Mothers will be informed that the program aims at improving early vaccination coverage for newborns, and that they will receive a small reward (2 cedis) if their baby receives both the BCG and the first dose of polio vaccination within the first two weeks of newborn life.

Mothers will be informed that if they decide to participate, the volunteer may contact them to encourage them to vaccinate their newborn and to document the vaccination if the woman reports that one or both vaccinations have been completed. Volunteers may contact enrolled woman via phone voice message, call, text or in person at the household to provide information about early vaccination and to encourage the woman to vaccinate her child.

If the woman reports to the volunteer that she has completed the vaccinations, the volunteer will document vaccination status and dates of completion, take a photo of the vaccination card and/or record the date and location that each of the vaccinations was received. The volunteer will not conduct any further follow-up with women who report completion of both early vaccines.

Volunteers will report the successful completion of both vaccinations along with the photo and/or location and date to the central study hotline for verification. As proof of completed vaccination, the volunteer will either need to submit a picture of the vaccination card, or provide the date and location of each vaccination to the project phone line for verification. If both vaccines were successfully completed within the first two weeks of newborn life, the mothers and community volunteers will each receive as small reward of 2 cedis (0.50 UDS).

Mothers in the incentives arm will not receive automated voice messages or automated SMS text reminders for vaccination.

The study hotline and management of the mobile system for sending automated voice messages and SMS texts to enrolled participants will be managed by the local implementing partner, Innovations for Poverty Action Ghana (IPA Ghana). The appointment of community volunteers in both treatment arms, management of informed consent documents and supervision of the list of enrolled participants, verification and distribution of incentives to volunteers for registering births and vaccination completion for woman in the incentives arm will also be managed by IPA Ghana.

Control arm

No study-supported activities will be conducted in control communities during the active intervention phase. Woman who give birth in these communities during the active intervention period will not be registered nor contacted by study volunteers nor receive study-generated reminders. No participants will be screened or enrolled in the control arm communities during the active intervention time period.

Endline Survey

To assess the reach of the program platforms as well as the relative effectiveness of the reminder and incentive-based activities, , we will conduct an endline survey at the end of the six month active study implementation phase, in each of the two intervention areas (10 communities each) and in the control area (5 communities) which did not receive any study-related interventions.

Surveyors will visit all households in the 15 communities and will identify households in which a woman who has given birth in the previous 12 months resides. If no household member is available at the time the surveyor visits, or a household member is available and reports that a birth occurred in the previous 12 months but that the mother is not currently available, the surveyor will return to the household up to two additional times to attempt to make contact. If any member of the household requests that the surveyor not return, this will be noted and no future visits will be conducted. If a potential participant or screened and enrolled participant requests that the surveyor returns at another more convenient time, the surveyor will return up to two additional times to attempt to complete the screening and enrolment or the interview process.

All women who reside in the community and have given birth in the previous 12 months will be eligible to participate in the endline survey. Among eligible woman, surveyors will describe the purpose and procedures of the survey and ask if the woman is available and interested in participating. If the woman is interested, the surveyor will guide her through the informed consent process. If the woman

is unavailable at this time, surveyors will return to the household up to two additional times to attempt to make contact and/or complete the survey. If the woman provides informed consent (see *Informed Consent Endline Survey*), the surveyor will engage in a brief interview with the woman using a structured survey tool, with data captured on an electronic tablet.

The survey will collect demographic and socioeconomic information about the household and the woman, basic information about the birth, vital status, and vaccination history of children. For each recent birth (within the previous 12 months), detailed vaccination reports will be collected. Vaccination history and date of birth will be based on nationally standardized health cards and vaccination cards if available, and maternal report if written documentation is unavailable. A modified version of the vaccination survey form developed for the Demographic and Health Surveys will be used to collect vaccination-related information. This vaccination survey module combines information from the child health card with maternal self-reports, and is widely used for national and international comparisons (see *Endline Questionnaire* attached).

Surveyors will be trained and sensitized regarding respectful and compassionate communication to prepare them for how to interact with a woman who reports the death of a newborn. Surveyors will refer woman who have lost a child to relevant local support resources as available and appropriate.

The interview will be conducted at the household at a room or location selected by the enrolled participant. Measures will be taken to attempt to identify a quiet and semi-private area free of distractions and with relative confidentiality. Surveyors will inform the woman that they are interested only in her experience and not the input of others and that there are no “right” or “wrong” answers to the survey. A woman may request to be accompanied during the interview by a friend, neighbor or household member of her choice if she so prefers. Surveyors will be conversant in local languages and the survey will be conducted in the language of choice of the respondent.

The survey will take approximately 10-15 minutes to complete. No costs will be incurred for enrolled woman for participating in the endline survey and no compensation or incentives will be offered.

Screening, enrollment, and data collection in the endline survey activity is independent of screening, enrollment and data collection in the intervention activities (reminders Group A and incentives Group B).

Primary and secondary outcomes

The primary study outcome is completion of early vaccination; we will consider a child as having successfully completed the early vaccination schedule if **the child has received the BCG vaccine <u>and</u> at least one dose of the polio vaccine within four weeks (28 days) after delivery**. We will additionally report the vaccine-specific coverage and the average age at vaccine receipt for each vaccine in each of the three arms.

The secondary outcome of the study will be the percentage of births identified via endline household survey that are reported by study volunteers. This outcome will be of particular interest for Ghana’s Birth and Death Registry, which is currently making major efforts to increase registration coverage in this region.

Expected Outcomes of the Study

This is a pilot investigation study. We primarily hope to inform estimates of the potential impact of mobile-phone based nudging systems for vaccines. If successful, similar programs would ideally be tested more rigorously through a larger study, or directly be incorporated into government programs.

Sample Size Calculation

According to the latest Demographic and Health Survey from Ghana, about 50% of children in the Northern region receive both Bacillus Calmette–Guérin (BCG) and Polio vaccines within the first 30 days after delivery. We hypothesize that this proportion can be increased to 80% with either intervention

(reminder-only and incentives). Assuming 20 children in each cluster, and an intra-class correlation coefficient (rho) of 0.05, a sample size of 100 children is required in each active treatment arm (reminder-only and incentives) and in the control communities for comparison, to achieve power 0.9 at alpha 0.05. With a crude birth rate of 40, we expect 20 births in each community within six months of baseline for the average village with a population of 1000, which means that the targeted sample size for the active treatment program (100 per active treatment arm) should be achieved in less than six months.

The endline survey will be conducted in all communities in the three arms and will attempt to enroll all women who delivered a newborn in the previous 12 months. This will additionally allow us to compare outcomes of interest across treatment groups and in the pre- initiation and study intervention periods. We anticipate this will include approximately twice the number of births expected in the 6-month intervention period (600). To account for woman who may have been missed during the intervention period, refused to participate, or moved into the community after initiation, we propose a maximum of 750 woman for the endline survey.

Sample Selection and Randomization

We will identify 15 villages in the Northern region of Karaga District, Ghana for the study. The specific communities engaged in the study will be selected based on a list of eligible communities within the District that will be made available to the research team after ethical approval is granted. Selection criteria will include the following (as available): population size (1000-20000 people), childhood vaccination coverage, facility delivery coverage, distance from/access to health facilities, existence of current maternal/newborn/child health services, and permission and willingness for engagement of local stakeholders. To avoid spillovers, a minimum distance of 5km between any two study villages will be enforced. The information to inform selection criteria will be made available by the District only after ethical approval is ascertained.

Random assignment to control and the two intervention arms will happen at the village level. Max-min randomization [13] will be used to ensure balance across the three intervention groups with respect to location, population size and other available demographic information.

Study Duration and Follow-Up

The two interventions will be implemented for six months in 10 pre-selected villages. During the six-month intervention period community volunteers will be engaged in recording all births in the catchment areas of the study communities.

The duration of participation for enrolled participants in each of the intervention arms will be approximately two weeks (soon after birth through two weeks of newborn life), with possible limited follow-up through 30 days of newborn life at the latest.

Reminder arm (Intervention Group A): Enrolled mothers will receive up to four voice or text messages to remind them of the importance and recommended timing of newborn vaccinations, starting from soon after birth and continuing through the first 10 days of newborn life (at approximately birth, 3, 7 and 10 days of life as appropriate). All contact with mothers will be completed by 30 days of newborn life at the latest.

Incentive arm (Intervention Group B)**:** Enrolled mothers will receive contact and encouragement from community volunteers to complete newborn vaccinations, and volunteers will contact women to attempt to document vaccination status starting from soon after birth and continuing through the first 10 days of newborn life, with possible follow-up for encouragement and verification. All contact with mothers will be completed by 30 days of newborn life at the latest.

After the intervention period ends in both active treatment arms (30 days after the enrollment of the last participant in each of the 2 active intervention arms), an endline survey will be conducted in all 15 communities (10 intervention arm communities and 5 control arm communities).

Endline survey: During the endline survey period all households in the communities will be visited by a household surveyor over an estimated two-month period. Each interview is expected to take approximately 10-15 minutes.

No further contact with subjects in any of the three arms will be conducted after the completion of the endline survey.

Duration of the Project

The total estimated project time is 18 months. We anticipate 6 months for study setup, six months for active intervention, 2 months to complete the endline survey, and 4 months for analysis.

Project Management

The overall project is managed by the PI, Dr. Günther Fink, and Dr. Gillian Levine at the Swiss TPH, Switzerland, in close collaboration with Innovations for Poverty Action Ghana, who will be in charge of all field-work related activities in Ghana. The Swiss TPH team will set up a weekly call with the project team, and will directly support IPA during the initial implementation and final data collection phases.

Informed Consent

Permission to conduct the study in each community will be sought from community leadership prior to the start of the study. Communities that meet specified criteria as summarized below will be considered for inclusion. The 15 communities will be selected from the list of eligible communities in the district. We already have approval from the district to work there (see Appendices). The study will only be carried out in selected communities in which community stakeholders have given the research team verbal permission to do so. The purpose and details of the study will be clearly explained to the community leaders and all questions they have will be adequately answered.

Informed consent will be sought from all eligible mothers for each of the active intervention arms programs (to participate in receiving SMS/voice reminders or being eligible to receive rewards) and additionally from all mothers eligible to participate in the endline survey. That is, woman identified at their households who have given birth in the past12 months at the time of the endline survey in all three study arm communities, regardless of participation in the intervention programs. Participants will be screened and enrolled separately in intervention procedures and in the endline survey (in those communities in which the intervention programs are delivered), and can choose whether to participate or not participate in each of these activities separately.

All three consent forms (endline survey, reminder-only intervention, incentives intervention) are attached. For mothers under 18 years at the time of screening and enrollment, permission to participate in the study (in intervention or in the endline survey) will be sought from a legal representative in the household.

Prior to participation in the study, the surveyors (endline survey) or community volunteers (reminder arm Intervention A and incentives arm Intervention B) will read the consent form to the respondent. The consent form will cover the purpose of the study, details of voluntary participation and the right to stop at any time, risks and benefits of participating, confidentiality of information, and contact information of the research team. Consent forms will be translated into the local language on the fly by enumerators and respondents will be explicitly informed about their rights as participants; namely that their participation in the survey is voluntary and that they may opt to discontinue participation at any time without it affecting them or their community in any way. Respondents who can read will be given the opportunity to read the consent form, and all potential participants will be given the opportunity to ask questions, and given satisfactory responses to their questions. Respondents will indicate their consent by writing their signature or thumb printing on the consent form. Paper copies of consent forms will be maintained by surveyors and community volunteers in the field during data collection. At the completion of active intervention field work all hard copies will be given to IPA to maintain in their central offices. Consent forms will be

kept separately from study data and will be kept in locked cabinets only accessible by relevant research staff.

The following processes will be thoroughly observed by this research team;

Confidentiality, Data Management and Data Security: Each field surveyor will sign a confidentiality sheet with IPA and will be trained in data security, human subjects, and proper treatment/engagement with the research subjects, including preparedness for adverse events. No information will be collected from respondents unless they express interest and confirm agreement to participate by giving consent. All participants will have the right to decline participation in the study. Data will be collected by local field surveyors who will travel to the home of respondents to conduct interviews.

For the endline survey, the field staff will record data electronically using password-protected tablets. As soon as possible, all the data will be uploaded onto a secure server and deleted from the tablet. Data from the server will be downloaded into encrypted folders. Only the research team will have access to the encrypted folders. Before analyses, personally identifiable information (PII) will be removed from the data and the data will be fully de-identified (a numeric code will be used to track each respondent). Participants will not be identified in any reports or publications that come from the research.

Vulnerable/ High Risk Group: Participation in this study will be strictly voluntary. Respondents will have the right to withdraw from the study at any time they so wish. The respondents to this study may include children (adolescents less than 18 years). Because this is a vulnerable population, we will seek the consent of their adult legal representative within their household.

Compensation: Study participants and community volunteers will not incur direct costs as the result of participation in this study. The recommended vaccinations are available free of charge to all woman/newborns in local health centers and the amount of time required for participation by enrolled participants in minimal. Therefore, compensation will not be provided. Some participants to this study will receive small incentives in the form of mobile cash money. No financial or non-monetary payments, rewards or incentives, nor compensation for costs incurred, will be provided to participants as part of the endline survey data collection activities.

Benefits: The children of participants in the intervention communities who are encouraged/reminded/incentivized to get vaccination will benefit from such vaccinations if they get them. However, we do not anticipate any significant benefits to the subjects participating in the study as respondents to the endline survey alone. We do anticipate benefits to society in the long run if either of the two pilot programs is successful in increasing early vaccination coverage.

Ethical Considerations

We follow the guidelines set in the Declaration of Helsinki. We anticipate minimal risks to study participants. The vaccinations promoted are recommended both by the WHO and Ghana Health Services, and are freely available at local clinics to all mothers regardless of study participation or treatment allocation arm. No costs will be incurred to participants in any of the study arms or as part of the endline survey as the result of their participation, and thus no compensation for expenses or costs will be provided to subjects in any of the arms.

Reminder arm (Intervention arm A): Participants may find the information or reminders sent to their phone disruptive or annoying. Participants will be informed that they can request to stop receiving reminders at any time with no penalty. There is a small chance that someone could steal information about who participated. However, the information collected is minimal and would cause minimal harm to participants if inadvertently made available. It is also possible that contact information of enrolled participants could be stolen. Participant contact information will be carefully guarded all times and will not be shared outside of the program and research team.

Incentive arm (Intervention arm B): Participants may find the encouragement from community volunteers disruptive or annoying, or may feel unduly pressured to receive vaccinations. Participants will be informed that they can request to stop receiving contact from the volunteer at any time with no penalty by calling the project phone number at no cost. Community volunteers will be instructed clearly not to exert undue pressure or coercion on mothers. To avoid possible coercion, the incentives to the woman for vaccination and to the volunteers for birth registration and vaccination documentation will be minimal. The incentive amount of 2 cedis was selected based on recommendations from local stakeholders of what an appropriate amount would be in this setting.

Control arm: The individuals living in the control arm will continue to receive the current standard of care in Ghana. The interventions being tested do not have strong existing evidence of effect and thus there is equipoise in continuing to offer standard of care in this setting.

Endline survey: The endline questionnaire will be very simple and will only include questions on basic demographics, birth history and vaccination status of children. It is possible that a woman may feel uncomfortable answering some of these questions. Interviewers will inform woman that they don't have to answer any question they don't feel comfortable answering. Interviewers will attempt to conduct the interview is a setting that is semi-private so that the woman doesn't feel uncomfortable answering around others and isn't influenced by other household members or community members. However, the information collected in the survey does not include sensitive information and we believe it would be unlikely to cause significant harm or bring stigma to a woman if it was accidently overheard by people in the household or community. It could be emotionally upsetting for a woman who has lost a child to be asked about the child and their medical care. Interviewers will be trained in respectful and compassionate interaction and refer woman to local support resources as available. The endline interview is anticipated to take no more than 15 minutes per household.

Problems Anticipated

This study involves minimal risks to subjects as described above.

One concern is that community volunteers may not report a sufficient number of births, so that reminder systems may not reach a sufficient number of women. We will closely monitor the monthly birth reports and will call or visit community volunteers in case birth reports are low or discontinued. A second concern is that mothers may not receive messages, or not listen to them. We will monitor the delivery status of messages carefully, and will coordinate with community volunteers if technical problems persist.

In both active treatment arms, it is possible that women could confuse the volunteers and message systems as mechanisms to access medical advice or medical care for newborns. We will inform women that volunteers and study staff running the reminder systems are not health professionals and that if they have a question or concerns about their baby's health they should visit a health facility.

It is also possible that participants in the treatment arms could mistakenly assume that the reminders or encouragement from volunteers cover all recommended care for their newborns and that if they complete the early vaccinations they have completed all recommended newborn care. We will inform all participants that there are other important services and vaccines that a baby should receive and that they should continue to seek care for the baby for other services and vaccines that are recommended.

In terms of potential adverse effects, one concern is that community volunteers may become too "enthusiastic" about the program, and exert too much moral pressure on mothers to get vaccinated. We will highlight this concern in our training. Only woman will be invited to be volunteers to reduce potential pressure from male community members on female study participants.

One additional potential problem is that even if women visit their local health facility to request vaccinations for their children, the vaccinations may not be available or not be offered at the time point

when they visit. We will work closely with the local government health officials and health facilities to trouble-shoot and avoid these possible problems.

Study Documents: Translations - Reference language

All of the master documents – including protocol, informed consent forms and study survey questionnaire – are in English. Translations to local language will be made on the fly by surveyors and volunteers, who will agree on standardized translations during training. Translation on the fly is standard in Ghana's Northern region because few people are able to read and write in the local language.

Ethical Approvals

Ethical approval for this project will be sought both in Switzerland (EKNZ) and in Ghana (Ghana Health Services).

Data Management and Statistical Analysis

All data will be captured electronically, and processed in the IPA Ghana office. Data will be stored at a password-protected and encrypted data server. Ongoing day-to-day data management and quality assurance will be conducted by IPA. Data will be shared with the PI (Dr. Fink) and his research team members at Swiss TPH during data collection for intermittent quality assurance purposes, and at completion of field work a de-identified dataset will be shared for involvement in final data cleaning and analysis. De-identified data will be kept by the research team indefinitely for use in future analyses as appropriate. Links between personally –identifiable information and study data will be destroyed after the primary data analysis for the study is complete.

Given the randomized assignment of treatment, it is anticipated that statistical analysis will be relatively straightforward. Logistic regression models will be used to analyze the primary and secondary outcomes of interest. Standard errors will be adjusted for cluster-level correlation using generalized estimating equations with robust variance-covariance estimation [14, 15].

Quality Assurance

All of the field work as well as compliance will be closely monitored by a project manager hired through the project partner, IPA Ghana. Interviewers will be supervised by a trained field supervisor, and a 10% subsample of households will be revisited for a short audit for the endline survey. sDr. Gillian Levine, will engage with the project manager to support data and research quality assurance activities. The study PI Dr. Fink will provide overall project oversight and supervision.

Dissemination of Results and Publication Policy

The results of the study will be shared through a peer-reviewed article as well as through the communication offices of IPA and the Swiss TPH.

I hereby certify that all of the information above is correct.

Günther Fink

Basel, August 9, 2018

References

1. Hughes MM, Katz J, Englund JA, Khatry SK, Shrestha L, LeClerq SC, et al. Infant vaccination timing: Beyond traditional coverage metrics for maximizing impact of vaccine programs, an example from southern Nepal. Vaccine. 2016;34(7):933-41. Epub 2016/01/21. doi: 10.1016/j.vaccine.2015.12.061. PubMed PMID: 26788880; PubMed Central PMCID: PMCPMC4744084.
2. Hamborsky J KA, Wolfe C. Epidemiology and prevention of vaccine-preventable diseases. 13th ed. . Washington, D.C.:: Public Health Foundation; 2015.
3. Ghana Statistical Service (GSS), Ghana Health Service (GHS), ICF International. Ghana Demographic and Health Survey 2014. . Rockville, Maryland, USA: GSS, GHS, and ICF International; 2015.
4. Raifman JRG, Lanthorn HE, Rokicki S, Fink G. The Impact of Text Message Reminders on Adherence to Antimalarial Treatment in Northern Ghana: A Randomized Trial. PloS one. 2014;9(10):e109032. doi: 10.1371/journal.pone.0109032.
5. Banerjee AV, Duflo E, Glennerster R, Kothari D. Improving immunisation coverage in rural India: clustered randomised controlled evaluation of immunisation campaigns with and without incentives. BMJ (Clinical research ed). 2010;340:c2220. Epub 2010/05/19. doi: 10.1136/bmj.c2220. PubMed PMID: 20478960; PubMed Central PMCID: PMCPMC2871989.
6. Kannisto KA, Koivunen MH, Valimaki MA. Use of mobile phone text message reminders in health care services: a narrative literature review. Journal of medical Internet research. 2014;16(10):e222. Epub 2014/10/19. doi: 10.2196/jmir.3442. PubMed PMID: 25326646; PubMed Central PMCID: PMCPMC4211035.
7. Horvath T, Azman H, Kennedy GE, Rutherford GW. Mobile phone text messaging for promoting adherence to antiretroviral therapy in patients with HIV infection. The Cochrane database of systematic reviews. 2012;(3):Cd009756. Epub 2012/03/16. doi: 10.1002/14651858.cd009756. PubMed PMID: 22419345.
8. de Jongh T, Gurol-Urganci I, Vodopivec-Jamsek V, Car J, Atun R. Mobile phone messaging for facilitating self-management of long-term illnesses. The Cochrane database of systematic reviews. 2012;12:Cd007459. Epub 2012/12/14. doi: 10.1002/14651858.CD007459.pub2. PubMed PMID: 23235644.
9. Gurol-Urganci I, de Jongh T, Vodopivec-Jamsek V, Atun R, Car J. Mobile phone messaging reminders for attendance at healthcare appointments. The Cochrane database of systematic reviews. 2013;(12):Cd007458. Epub 2013/12/07. doi: 10.1002/14651858.CD007458.pub3. PubMed PMID: 24310741.
10. Vodopivec-Jamsek V, de Jongh T, Gurol-Urganci I, Atun R, Car J. Mobile phone messaging for preventive health care. The Cochrane database of systematic reviews. 2012;12:Cd007457. Epub 2012/12/14. doi: 10.1002/14651858.CD007457.pub2. PubMed PMID: 23235643.
11. Odone A, Ferrari A, Spagnoli F, Visciarelli S, Shefer A, Pasquarella C, et al. Effectiveness of interventions that apply new media to improve vaccine uptake and vaccine coverage. Human vaccines & immunotherapeutics. 2015;11(1):72-82. Epub 2014/12/09. doi: 10.4161/hv.34313. PubMed PMID: 25483518; PubMed Central PMCID: PMCPMC4514191.
12. Oyo-Ita A, Wiysonge CS, Oringanje C, Nwachukwu CE, Oduwole O, Meremikwu MM. Interventions for improving coverage of childhood immunisation in low- and middle-income countries. The Cochrane database of systematic reviews. 2016;7:Cd008145. Epub 2016/07/11.

doi: 10.1002/14651858.CD008145.pub3. PubMed PMID: 27394698; PubMed Central PMCID: PMCPMC4981642.
13. Bruhn M, McKenzie D. In Pursuit of Balance: Randomization in Practice in Development Field Experiments. American Economic Journal: Applied Economics. 2009;1(4):200-32. doi: doi: 10.1257/app.1.4.200.
14. Wang M, Long Q. Modified robust variance estimator for generalized estimating equations with improved small-sample performance. Statistics in medicine. 2011;30(11):1278-91. Epub 2011/05/04. doi: 10.1002/sim.4150. PubMed PMID: 21538453.
15. Huang S, Fiero MH, Bell ML. Generalized estimating equations in cluster randomized trials with a small number of clusters: Review of practice and simulation study. Clinical trials (London, England). 2016;13(4):445-9. Epub 2016/04/21. doi: 10.1177/1740774516643498. PubMed PMID: 27094487.
